# Chromatin Profiles Are Prognostic of Clinical Response to Bortezomib-Containing Chemotherapy in Pediatric Acute Myeloid Leukemia: Results from the COG AAML1031 Trial

**DOI:** 10.3390/cancers16081448

**Published:** 2024-04-09

**Authors:** Anneke D. van Dijk, Fieke W. Hoff, Yihua Qiu, Stefan E. Hubner, Robin L. Go, Vivian R. Ruvolo, Amanda R. Leonti, Robert B. Gerbing, Alan S. Gamis, Richard Aplenc, Edward A. Kolb, Todd A. Alonzo, Soheil Meshinchi, Eveline S. J. M. de Bont, Terzah M. Horton, Steven M. Kornblau

**Affiliations:** 1 Division of Pediatric Oncology and Hematology, Department of Pediatrics, University Medical Center Groningen, University of Groningen, 9713 GZ Groningen, The Netherlands; fieke.hoff@utsouthwestern.edu (F.W.H.);; 2 Department of Leukemia, M.D. Anderson Cancer Center, The University of Texas, Houston, TX 78712, USA; 3 Department of Molecular Therapy and Hematology, M.D. Anderson Cancer Center, The University of Texas, Houston, TX 78712, USA; 4 Clinical Research Division, Fred Hutchinson Cancer Research Center, Seattle, WA 98109, USA; 5 COG Statistics and Data Center, Monrovia, CA 91016, USA; 6 Department of Hematology-Oncology, Children’s Mercy Hospitals and Clinics, Kansas City, MO 64108, USA; 7 Division of Pediatric Oncology and Stem Cell Transplant, Children’s Hospital of Philadelphia, Philadelphia, PA 19104, USA; 8 Nemours Center for Cancer and Blood Disorders, Alfred I. DuPont Hospital for Children, Wilmington, DE 19803, USA; 9 Keck School of Medicine, University of Southern California, Los Angeles, CA 90007, USA; 10 Texas Children’s Cancer and Hematology Centers, Baylor College of Medicine, Houston, TX 77030, USA

**Keywords:** pediatric acute myeloid leukemia, reverse-phase protein array, bortezomib

## Abstract

**Simple Summary:**

Bortezomib-containing chemotherapy did not improve the clinical outcome in the AAML1031 study in terms of overall survival and event-free survival compared to standard chemotherapy. We characterized epigenetically distinct proteomic profiles in a large cohort of pediatric patients that participated in this study using the reverse-phase protein array. We observed in the patient group that received standard therapy that a higher expression of 16 histone-modulating enzymes (HMEs) was an independent variable that predicted higher relapse risk three years after a second induction therapy compared to those with a lower HME protein expression. Also, there was significantly improved overall survival for those with a high HME expression who were treated with the bortezomib-containing chemotherapy, compared to high-HME patients treated without bortezomib. We also demonstrated that patients with a higher expression of HME had more open chromatin surrounding promoter sides compared to those with lower HME protein levels using ATAC-seq.

**Abstract:**

The addition of the proteasome inhibitor bortezomib to standard chemotherapy did not improve survival in pediatric acute myeloid leukemia (AML) when all patients were analyzed as a group in the Children’s Oncology Group phase 3 trial AAML1031 (NCT01371981). Proteasome inhibition influences the chromatin landscape and proteostasis, and we hypothesized that baseline proteomic analysis of histone- and chromatin-modifying enzymes (HMEs) would identify AML subgroups that benefitted from bortezomib addition. A proteomic profile of 483 patients treated with AAML1031 chemotherapy was generated using a reverse-phase protein array. A relatively high expression of 16 HME was associated with lower EFS and higher 3-year relapse risk after AML standard treatment compared to low expressions (52% vs. 29%, *p* = 0.005). The high-HME profile correlated with more transposase-accessible chromatin, as demonstrated via ATAC-sequencing, and the bortezomib addition improved the 3-year overall survival compared with standard therapy (62% vs. 75%, *p* = 0.033). These data suggest that there are pediatric AML populations that respond well to bortezomib-containing chemotherapy.

## 1. Introduction

Despite intensive chemotherapy and hematopoietic stem cell transplantation, mortality rates in children with acute myeloid leukemia (AML) remain high at 30% [1]. As AML is defined by a wide array of molecular events, including chromosomal translocations and driver mutations, that co-occur in various combinations, targeting AML therapy for individual leukemias can be challenging. Concurrent with the expansion in genetic characterization, there are a myriad of new therapeutic agents targeting many components of AML cell metabolism and physiology. Most of these new therapeutic agents target proteins, and since the net effect of molecular events is predominantly alterations in protein activation, this creates an unmet need of efficiently directing clinical agents for optimal personalized therapy [2,3]. We argue that a more complete understanding of the proteomic landscape in AML will contribute to better patient risk stratification and possibly improved targeted therapy selection [4,5].

The proteasome, a large multiprotein complex located in both the cytoplasm and nucleus, functions to regulate protein homeostasis by degrading damaged and un-needed proteins. In the nucleus, proteasome complexes are present at centromeres, near RNA-polymerase II complexes of actively transcribed genes and at sites with DNA damage, including telomeres [6]. It is thought that 26S proteasomes are recruited to the chromatin in response to transcription, for the degradation of ubiquitinated proteins at sites of highly active genes [7], and transcription complexes [8,9]. Additionally, proteasome inhibition alters the chromatin structure and transcriptional output [10], and Leshchenko et al. have shown that the proteasome inhibitor bortezomib causes a global loss of methylation [11]. Another study by Kikuchi et al. demonstrated earlier that bortezomib targets histone deacetylases (HDACs) [12]. Interestingly, the number and activity of proteasomes is elevated in AML, and a selective inhibitor of the 26S proteasome sensitizes AML blasts to standard induction chemotherapy agents in vitro [13,14]. In adult AML, adjuvant proteasome inhibition was shown to increase the percentage of patients reaching complete remission after induction, particularly in elderly AML patients [15,16]. When the 26S proteasome inhibitor bortezomib (Velcade, ‘BTZ’) was added to standard pediatric AML reinduction chemotherapy in a Children’s Oncology Group (COG) phase 2 clinical trial (AAML07P1), its use was considered safe and potentially effective for pediatric AML [17].

However, a randomized phase 3 clinical trial (AAML1031; NCT01371981) conducted by the COG revealed no differences in overall survival (OS) or event-free survival (EFS) between standard treatment (cytarabine, daunorubicin and etoposide, ‘ADE’) and ADE+BTZ when all patients were analyzed as one group [18]. From this phase 3 trial, we examined protein expressions from 483 AML patients and were able to identify protein expression signatures that clearly benefitted from the addition of bortezomib [19], suggesting that specific AML populations could benefit from proteasome inhibition. As the proteasome plays an integral role in nuclear events, particularly in the context of active chromatin, we hypothesized that histone and chromatin modifications would be associated with the response to bortezomib-containing chemotherapy. In this study, we examined the relationship between chromatin regulation and histone modifiers using reverse-phase protein arrays (RPPAs) and transposase-accessible chromatin using sequencing (ATAC-seq). We compared the differential response of ADE vs. ADE+BTZ to gain a better understanding of proteasome inhibition sensitivity in pediatric AML.

## 2. Materials and Methods

### 2.1. Patient Population

All patients were enrolled in the COG phase 3 AAML1031 clinical trial. In total, we analyzed 483 peripheral blood samples from de novo AML patients. The trial included a 1:1 randomization of cytarabine (ara-C), daunorubicin and etoposide (ADE) ± the proteasome inhibitor bortezomib (ADE+BTZ) at a dose of 1.3 mg/m^2^ on days 1, 4, 8 and 11 of each of four cycles. Patient samples were acquired during routine diagnostic assessments before treatment, and 10 and 24 h after the start of the induction therapy on “Day 1”. Samples were collected between July 2011 and February 2017 and written informed consent was obtained in accordance with the Declaration of Helsinki and local human protection boards according to institutional regulations. Outcome data were available for 410 of the 483 patients, and the data were frozen on 30 June 2019. More information about the patient population, choice of treatment, sample processing after collection and proteomic profiling of the patient samples using RPPAs can be found in Appendix A.

### 2.2. RPPA Methodology

The proteomic profiling of the AML patient samples was generated using an RPPA performed as previously described [4,19,20,21,22,23]. A more detailed description of the RPPA methodology is provided in Appendix A. In short, slides were probed with a total of 296 strictly validated primary antibodies. A secondary antibody was used to amplify the signal, and a stable dye for signal detection. After an initial analysis, there were protein levels normalized relative to the mean expression of non-malignant bone marrow CD34+ cells from 30 healthy donor samples (20 pediatric and 10 adult samples).

### 2.3. ATAC-seq

ATAC-seq was performed, as previously described, on 24 peripheral blast samples from patients enrolled in the AAM1031 clinical trial. Patient selection was based on HME expression patterns from the RPPA data. Based on the RPPA results, we aimed to assess the chromatin accessibility between patients with high histone-modifying enzymes (*n* = 15) and patients with low histone-modifying enzymes (*n* = 9) regardless of the histone modification mark expression profile. A detailed description of the procedure can be found in Appendix A.

### 2.4. Cell Lines and shRNA Knockdown Cells

OCI-AML3 cells were a kind gift from Mark Minden (Ontario Cancer Institute; Toronto, ON, Canada). THP-1 and HEK 293T cells were obtained from ATCC (Manassas, VA, USA). FOXO3A was knocked down through a lentiviral transduction of shRNA using pGIPZ based shRNAmiR transfer vectors (Open Biosystems, Huntsville, AL, USA). Concerning a negative control, we used pGIPZ non-silencing control (Open Biosystems). Details about the lentivirus production are provided in Appendix A, as well as details about cell treatment and cytotoxicity analysis.

### 2.5. RNA-seq

A gene expression analysis was performed on 390 of the 483 samples with RPPA data, as previously described [24]. A differential gene expression analysis of 21,927 analyzed genes was performed between patients with higher vs. lower FOXO3 protein expressions.

### 2.6. Statistical Analysis

Outcome data were available for 410 of the 483 patients and the data were frozen on 30 June 2019. The associations between protein clusters and clinical variables were calculated using a Chi-Square and Fisher’s exact test when data were sparse for categorical variables [25]. The Kruskal–Wallis test was used to test medians for continuous variables [26]. Analyses of overall survival (OS) and event-free survival (EFS) were conducted using the Kaplan–Meier method [27], and the log-rank test was used for comparisons [28]. OS was defined as time from study entry until death, and EFS was defined as the time from the end of induction until death, refractory disease or relapse [18]. Relapse risk (RR) was calculated using Gray’s test [28]. RR was defined as the time from the end of Induction II (for patients in CR) to relapse. Death without relapse was considered a competing event, as previously described [18]. The Cox proportional hazard model was used to estimate hazard ratios for univariable and multivariable analyses of OS and EFS, whereas competing risk regression was used for RR [29].

## 3. Results

### 3.1. Histone- and Chromatin-Modifying Protein Expressions Are Heterogeneous among De Novo Pediatric AML

**Figure 1:**
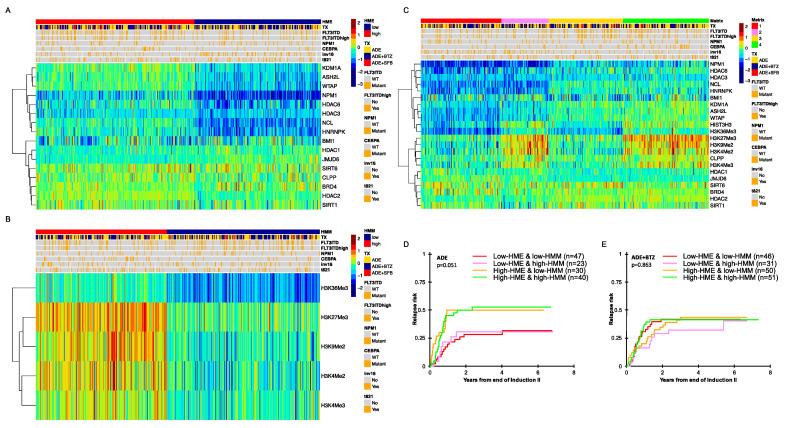
(**A**) RPPA-based heatmaps generated via progeny clustering showing the expressions of 16 histone- and chromatin-modifying enzymes (HMEs) and (**B**) 5 histone methylation marks in 483 pediatric AML patient samples along with received treatment (TX, ADE = ara-C, doxorubicin and etoposide, BTZ = bortezomib and SFB = sorafenib), cytogenetic aberrations and driver mutations. (**C**) RPPA-based heatmap of the 483 patients generated using the HME and HMM cluster status (red: low-HMM and low-HME, pink: low-HME and high-HMM, yellow: high-HME and low-HMM and green: high-HME and high-HMM) along with clinical features. (**D**) Relapse risk for patients in complete remission after two courses of induction therapy in ADE treated patients (*n* = 164). (**E**) Relapse risk for patients in complete remission after two courses of induction therapy in ADE+BTZ-treated patients (*n* = 210).

An RPPA was used to measure protein expressions of 16 histone- and chromatin-modifying enzymes (HMEs) and 5 histone methylation marks (HMM), along with 275 other proteins in a cohort of 483 pediatric AML patients that participated in the COG AAML1031 trial. As controls, non-malignant bone marrow-derived CD34+ cells from 30 healthy pediatric and adult donors were used. We hypothesized that there might be different patterns of epigenetic readers and writers (HMEs) that could yield similar patterns of histone methylation marks (HMMs), so we started by looking for recurrent protein expression patterns within each of the HME and HMM functional subsets using unbiased progeny clustering analysis [30]. Since the HMM status is the net consequence of epigenetic writers and readers actions (HMEs), we first analyzed the expression based on HMMs and HMEs separately. For HMEs (Figure 1A), the 16 member proteins formed two clusters (left side, *y*-axis dendrogram), with the expression of the top cluster (KDM1A, ASH2L, WTA1, NPM1, HDAC3, HDAC6, NCL and hnRNPK) dividing patients into those with a high expression, similar to that of the normal CD34+ cells (red cluster), or with a below normal expression (blue cluster). The HME proteins in the bottom half are generally overexpressed relative to the normal CD34+ cells and similarly expressed across the two patient groups, except for BRD4 (high in blue cluster) and HDAC2 (high in red cluster). The clustering of the HMMs cleanly dichotomized into two patient clusters, one with high levels of HMMs and the other with low levels of HMMs (Figure 1B).

A composite of both HMM and HME (Figure 1C) shows that expressions within the HMMs and HMEs are independent of each other as the size of the four groups are proportional to the product of the percentage in each individual group. To analyze whether there are interactions between the HME and HMM profiles and to investigate if these have clinical impact, we built a 4 × 2 matrix with four clusters for both treatment arms: C1 with low expressions for both HMMs and HMEs; C2 with a low expression of HME, but high for HMM; C3 with high HME, but low HMM, and C4 with a relative upregulation of HMM and HME (Figure 1C). When we compare these four groups among the 410 patients that had clinical data available (Table 1), no differences based on sex, age, race, ethnicity, CNS status, WBC, risk group, FLT3-ITD, NPM1 and most cytogenetic subsets were found. The exceptions were a higher percentage of inv(16) patients in C1 and C2 (*p* = 0.02) and significantly more abnormalities in chromosome 5, 7 and 8 in C3 (14%) (*p* = 0.04). CEBPA mutations were more frequent in C4 (17%) compared to C1 (4%), C2 (10%) and C3 (7%) (*p* = 0.01). Patients with a high-allelic ratio (>0.4) of the FLT3-ITD mutation were more frequent in C3 (24% vs. 10%, 10% and 13% in C1, C2 and C4, respectively, *p* = 0.01). Therefore, patients defined as “high risk” according to the AAML1031 study guidelines [31] were statistically more abundant in C3 (41%, *p* = 0.02).

### 3.2. Pediatric AML with High HME Levels Benefitted from Bortezomib-Containing Chemotherapy

When outcomes based on the matrix status were assessed, we observed that patients with low HME levels treated with ADE (*n* = 164) had a lower RR compared to those with high HME levels (28% and 30% vs. 50% and 53%, *p* = 0.051, Figure 1D), regardless of the HMM status. In contrast, the ADE+BTZ patients with high HME levels had similar outcomes to those with low HME levels (Figure 1E, *n* = 210, *p* = 0.863).

**Figure 2:**
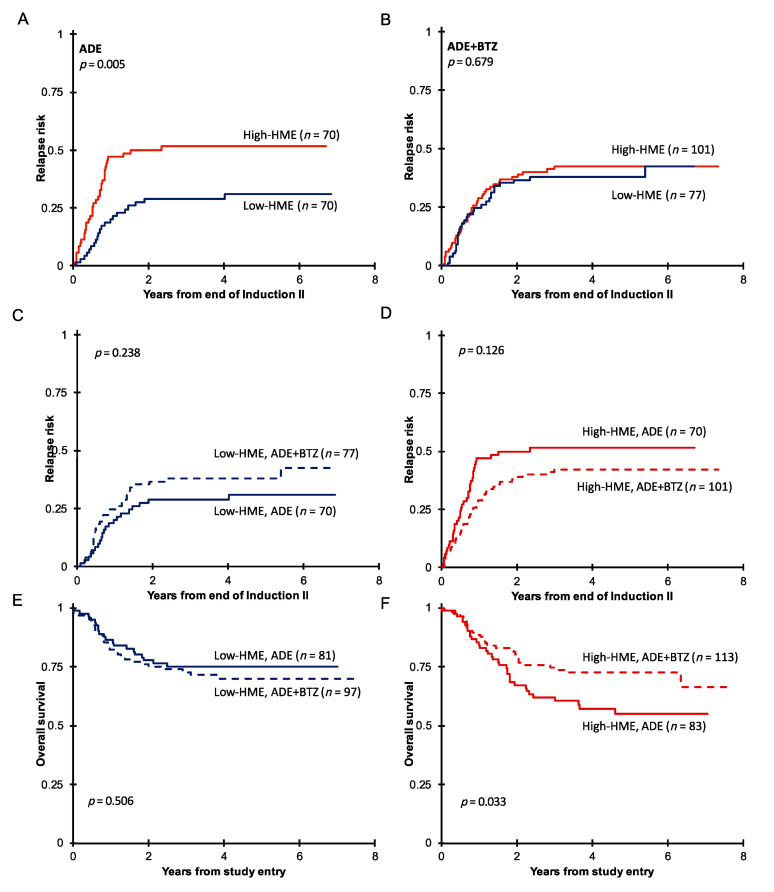
(**A**) Remission duration after two courses of induction therapy with ADE (*n* = 164) or (**B**) ADE+BTZ (*n* = 210) in treated patients with high HME levels (red) compared to those with low HME levels (blue). Remission duration after two courses of induction therapy in (**C**) the low-HME and (**D**) high-HME populations separately in ADE vs. ADE+BTZ treatment regimes. Overall survival in (**E**) low-HME and (**F**) high-HME profile patients in ADE vs. ADE+BTZ.

When the outcome with the HME clusters was assessed in context of the treatment group, there were significant differences in the outcome. In the ADE-treated cohort, those with high HME levels had a significantly higher relapse rate (Figure 2A, *p* = 0.005, 3-year RR from end of Induction II; high-HME = 52% vs. low-HME = 29%) and an inferior overall survival (Figure 2E,F, *p* = 0.028, 3-year survival low-HME 75% vs. high-HME 62%). In those treated with ADE+BTZ the higher relapse rate was comparable to that of the low-HME cases (Figure 2B, *p* = 0.679, 3-year RR, high-HME = 42% vs. low-HME = 38%), and the OS was similar as well (Figure 2E,F, *p* = 0.689, 3-year survival low-HME 73% vs. high-HME 75%). Thus, the negative prognostic impact of high HME levels was negated through the addition of PI. No significant differences were found between ADE- and ADE+BTZ-treated patients in terms of RR in the low- or high-HME group (Figure 2C,D), but 1 year after the end of the second induction cycle, RR was 47% in the high-HME ADE-treated group and only 29% in the high-HME group who received ADE+BTZ (Figure 2D). Also, there was a significantly improved 3-year OS for those with high HME levels who were treated with bortezomib-containing chemotherapy compared to those treated with ADE alone (62% vs. 75% *p* = 0.033, Figure 2F). The significant difference in OS therefore results from the combination of the non-significant trend for a shorter remission duration and the non-significant trend for a higher relapse rate in the ADE treatment arm.

### 3.3. High-HME Proteomic Profile Is an Independent Adverse Prognostic Factor for Relapse in ADE-Treated De Novo Pediatric AML

The C3 (high HME and low HMM levels) and C4 (high HME and high HMM levels) memberships were both independently correlated with higher RR after two courses of ADE, indicating that the HME cluster membership was prognostic regardless of the HMM expression. Indeed, a low-HME status was a univariable predictor for improved OS 3 years post diagnosis (HR = 0.54, 95%CI = 0.31–0.94, *p* = 0.03) and RR (HR = 0.47, 95%CI = 0.28–0.80, *p* = 0.005, Table 2) after ADE. As there were some imbalances in the distribution of other clinical features with known prognostic impact between the low- and high-HME clusters, we asked if the HME status was an independent predictor of outcome. In a multivariable risk regression analysis, the HME status was an independent variable for RR (HR = 0.45, 95%CI = 0.26–0.77, *p* = 0.004, Table 3) but not for OS.

### 3.4. ATAC-seq Reveals Higher Chromatin Accessibility in Patients with More Activated HME

**Figure 3:**
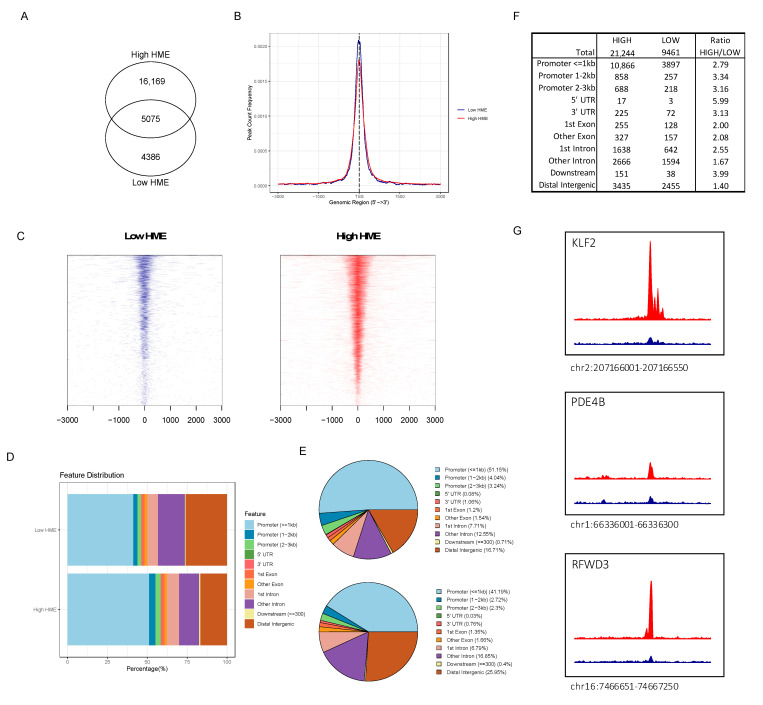
(**A**) Open chromatin regions identified in high-HME (21,244) vs. low-HME (9461) expressors with 5075 overlapping regions; (**B**) region count frequency surrounding transcription start site (TSS) in high-HME (red) vs. low-HME (blue) expressors; (**C**) heatmap of open chromatin to TSS regions for low-HME (blue) and high-HME (red) expressors; (**D**) visualization of genomic annotation of accessible chromatin in low-HME and high-HME expressors; (**E**) pie plot visualization of genomic annotations of open chromatin in high-HME (upper panel) vs. low-HME (lower panel) expressors; (**F**) absolute number of open chromatin region annotations and ratios between low- and high-HME expressors; (**G**) examples of genomic tracks that were enriched in high-HME (red) compared to low-HME (blue) expressors.

We hypothesized that the differences between a low and high-HME status would result in global modifications to the epigenome that would have the consequence of modulating the protein expression. To look for evidence of this effect on chromatin, we compared genomic chromatin accessibility between patients with either high or low HME expression levels. We performed ATAC-seq to explore active (i.e., open) and inactive (i.e., condensed) chromatin in leukemic blasts in a total of 24 cytogenetically normal samples, 9 low-HME and 15 high-HME AML patients. The ATAC-seq results were of high quality as assessed using short fragment lengths (Figure S1) and high mapping rates (>98.5%). Instead of peak calling for the high read density, we used a window-based strategy to summarize read counts across the genome (details can be found in Appendix A). In total, we identified 18,103 filtered windows across all leukemic samples with a low HME status (*n* = 9) and 84,178 in the samples with high HME levels (*n* = 15, Appendix A). After non-linear loss normalization, windows were clustered into regions. Genomic enriched windows less than 100 bp apart were considered to be adjacent and were grouped into the same cluster (‘region’). For the low-HME expressors, 9461 open chromatin regions were identified, and 21,244 open regions were identified across all high-HME samples. An overlap of 5075 genomic regions was identified between low- and high-HME expressors (Figure 3A). A similar enrichment of open chromatin was observed at transcription start sites between low- and high-HME expressors (TSS, Figure 3B,C). A total of 58% of the identified open regions in the high-HME expressors were found surrounding promoters (3 kb) compared to 46% in the low-HME expressors (Figure 3D,E). High-HME expressors showed a 2.8 absolute upturn in regions <1 kb from promoters compared to those with a low HME expression, but the absolute rise in distal intergenic regions was only 1.4 (Figure 3F). Among the identified differential accessible regions, 171 genomic regions were significantly more accessible in all high-HME expressors compared to low-HME expressors (Table S1). Three examples of these regions are shown in Figure 3G.

### 3.5. Activated Histone Deacetylase Proteins Associated with Loss of Transcription Factor FOXO3

**Figure 4:**
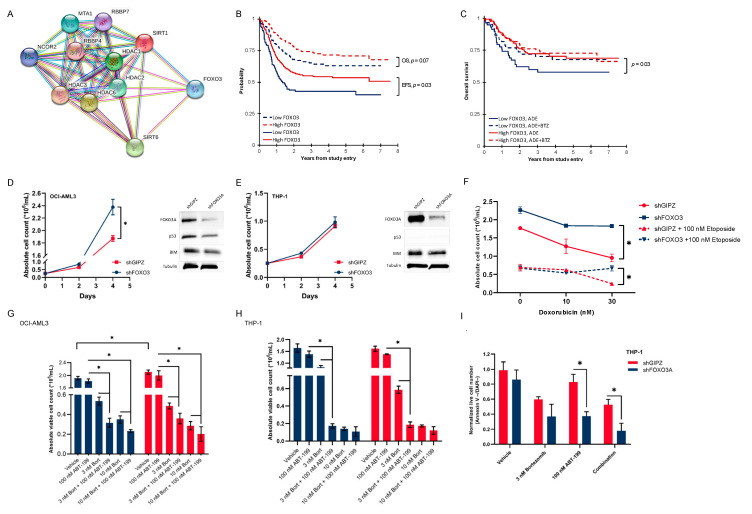
(**A**) STRING network analysis for proteins that correlated with histone deacetylase (HDAC1, HDAC2, HDAC3, HDAC6, SIRT1 and SIRT6) revealed an association with transcription factor FOXO3; (**B**) overall survival (OS) and event-free survival (EFS) according to FOXO3 stratification in pediatric AML (*n* = 410); (**C**) OS according to FOXO3 stratification (low FOXO3 levels, blue and high FOXO3 levels, red) and treatment group (ADE, solid lines and ADE+BTZ, dashed lines); (**D**) growth curves of FOXO3 knockdown in p53-^WT^ OCI-AML cells (shFOXO3, blue) compared to controls (shGIPZ, red) (day 4, *p* = 0.004) along with Western Blot showing protein expressions of FOXO3A, p53, BIM and Tubulin as loading control in shFOXO3 vs. shGIPZ at baseline; (**E**) growth curves of FOXO3 knockdown in p53-^null^ THP-1 cells (shFOXO3, blue) compared to controls (shGIPZ, red) along with Western Blot showing protein expressions of FOXO3A, p53, BIM and Tubulin as loading control in shFOXO3 vs. shGIPZ at baseline, the uncropped blots and the densitometry readings/intensity ratio of each band are presented in Figure S8; (**F**) THP-1 shFOXO3 cells were more resistant to doxorubicin and a combination of doxorubicin and etoposide after 72 h compared to control (shGIPZ); (**G**) viable cell count without (Vehicle) with a single bortezomib (Bort) and venetoclax (ABT-199) treatment and in combination with shFOXO3 and shGIPZ OCI-AML3 cells after 72 h; (**H**) viable cell count without (Vehicle) with single bortezomib (Bort) and venetoclax (ABT-199) treatment and in combination with shFOXO3 and shGIPZ THP-1 cells after 72 h; (**I**) Annexin V and DAPI staining (normalized live cell number) in shFOXO3 and shGIPZ THP-1 cells after single 3 nM bortezomib and 100 nM ABT-199 treatment as well as in combination after 72 h. * *p* value < 0.05.

We then aimed to identify known proteins that are associated with HMEs. Since HDAC inhibitors are used in combination with bortezomib in certain groups of multiple myeloma patients, we aimed to investigate a protein–protein interaction analysis using HDACs on our panel. The analysis was performed using STRING (https://string-db.org) with an interaction score of high confidence > 0.700, and it revealed an association between the 6 HDACs (HDAC1-3, HDAC6, SIRT1 and SIRT6) and the transcription factor FOXO3 (Figure 4A). This captured our interest since SIRT1 promotes FOXO3 ubiquitination and subsequent proteasome-dependent degradation [32]. In our pediatric AML cohort, FOXO3 was expressed at a significantly lower level in patients with high HME levels compared to those with low HME levels (Figure S2, *p* < 0.001). Based on this finding, we were eager to examine the clinical relevancy of FOXO3 in our cohort, and its relations to bortezomib.

### 3.6. Pediatric AML Patients with Low FOXO3 Levels Are Potential Candidates for Bortezomib Addition

Cox regression for survival analysis revealed that the expression of FOXO3 was prognostic for EFS in both univariable (HR = 0.60, 95%CI = 0.37–0.97, *p* = 0.038) and multivariable (HR = 0.61, 95%CI = 0.37–0.99, *p* = 0.044) analysis (Table S2). We then divided the 374 patients who received ADE or ADE+BTZ (excluding those who received sorafenib [SFB]) into two clusters (low [*n* = 106] and high [*n* = 268] FOXO3 expressions using the median expression in normal CD34+ as the cut-off) to examine the FOXO3 expression in relation to outcomes. A Kaplan–Meier survival analysis showed a trend toward poor OS (3-year OS of 65% vs. 74%, *p* = 0.07) and significantly poor EFS overall (3-year EFS of 43% vs. 54%, *p* = 0.03) in low-FOXO3 expressers compared to patients with a high FOXO3 expression (Figure 4B). However, the poor prognostic effect of low FOXO3 levels for OS was exclusively seen in the ADE-treated cohort (3-year OS of 58% vs. 73%, *p* = 0.03), but not in that treated with ADE-BTZ (3-year OS of 70% vs. 75%, *p* = 0.52) (Figure 4C).

### 3.7. Low-FOXO3 Cells Show Resistance to Doxorubicin and Etoposide In Vitro

To validate our clinical findings, we performed an in vitro knockdown of FOXO3 using a short hairpin construct in OCI-AML3 (p53^WT^) and THP-1 (p53^null^) cell lines [33,34]. The shFOXO3 OCI-AML3 cells had a short-term growth advantage compared to controls (shGIPZ, day 4, *p* = 0.004, Figure 4D), but not the shFOXO3 THP-1 cells compared to the controls (Figure 4E). To study differences in cell proliferation between shFOXO3 cells and controls upon treatment, we performed cell viability and apoptosis assays. Apoptosis of leukemic cells in response to doxorubicin has been shown to be dependent upon FOXO activation [35]. In THP-1 cells (p53^null^), we observed that shFOXO3 cells were slightly more resistant to doxorubicin and etoposide combination therapy than controls (*p* = 0.03, Figure 4F). A similar decrease in viable cell number were seen in bortezomib-treated cells with and without the knockdown of FOXO3 (Figure S3).

### 3.8. In Vitro Proteasome and bcl-2 Inhibition Are More than Additive, and This Effect Is Dependent on the FOXO3 Protein Expression

In adults, therapeutic regimes in AML frequently include bcl-2 inhibition with venetoclax (ABT-199). Since FOXO3 acts as transcriptional regulator of several pro-apoptotic proteins in the bcl-2 family, including Fas ligand, TRAIL and BIM, we wanted to test whether bortezomib and venetoclax enhanced cytotoxic effects, and if this was related to the FOXO3 expression. To examine this interaction, THP-1 cells were treated with each agent singly and in combination. After 72 h of treatment, a low dose of either BTZ or venetoclax did not reduce cell numbers in either shFOXO3 or control transfected cells (shGIPZ). Combination therapy, however, effectively reduced cell count and viability in both OCI-AML3 and THP-1 cells regardless of the FOXO3 status (*p* < 0.001, Figure 4G,H). Flow cytometry using Annexin V staining revealed a significantly higher apoptotic proportion in shFOXO3 THP-1 cells compared to control cells after single venetoclax and combination therapy with venetoclax and BTZ (*p* < 0.001, Figure 4I).

### 3.9. FOXO3 Negatively Correlates with the Vimentin Expression on the mRNA and Protein Levels

To gain a better understanding of why low-FOXO3 expressors do poorly in pediatric AML, we assessed differentially expressed genes (DEGs) through mRNA-seq analysis using FOXO3 stratification. We identified 4685 significant DEGs between low- and high-FOXO3 patient groups. Genes were considered differentially expressed at FDR-adjusted *p*-value less than 0.05 (Figure S4A). Using pathway topology analysis (SPIA) [36] to take into account gene interaction information along with the fold changes and adjusted *p*-values, we identified affected pathways between the low- and high-FOXO3 expressors. SPIA calculates dysregulated pathways based on over-representation and signaling perturbations accumulation using the KEGG database. Among the 137 pathways in the KEGG database, *Focal adhesion* and *ECM-receptor interaction* had lower FDR and Bonferroni adjusted global *p*-values than 0.05 (Figure S4B,C) and were considered dysregulated pathways according to FOXO3 in our cohort. Both pathways affect cell adhesion, migration, proliferation and apoptosis. A gene involved in cell adhesion that was inversely correlated with FOXO3 on the mRNA level was vimentin. We also found that the FOXO3 protein had the strongest negative correlation with vimentin on the RPPA (r = −0.47, *p* < 0.01, Figure S5A,B).

In leukemia, it has been speculated that vimentin acts as a negative regulator of apoptosis and confers increased stress resistance, promoting cell survival [37]. However, we observed that ADE patients were prognostically stratified by FOXO3 regardless of vimentin levels (Figure S5C,D). When comparing ADE and ADE+BTZ in FOXO3–vimentin subgroups, the bortezomib addition had the largest beneficial effect in patients with low FOXO3 and high vimentin levels (3 years OS of 57% vs. 81%, *p* = 0.006, Figure S5E).

### 3.10. Characterization of an Open Chromatin Signature for High-HME Patients That Responded to Bortezomib-Containing Chemotherapy

To gain a better understanding of why patients with high HME levels benefitted from the bortezomib-containing chemotherapy, we sought a unique open chromatin signature for high-HME patients using the ATAC-seq data. We used *csaw* to count reads across the genome. A total of 50,397,703 regions were identified using the sliding window approach. These were filtered by local enrichment, resulting in 139,798 regions used for differential accessibility (“DA”) analysis. After normalization, the data were transformed into log2 counts per million using the limma-voom method (Figure S6A) [38,39]. To obtain the unique open chromatin signature for high-HME patients that benefitted from bortezomib-containing chemotherapy, we compared accessible regions between high-HME patients that responded to ADE+BTZ with low-HME patients that relapsed after the same treatment. After formulating these contrasts, we fitted the linear model to the data (Figure S6B) and tested for DA regions (Figure S6C). Detailed descriptions of the filtering, normalization and DA analysis are provided in Appendix A. A comparison of high-HME classified patients that responded to ADE+BTZ treatment vs. low-HME patients that relapsed after the same treatment identified 572 upregulated regions in high-HME responders and 583 upregulated regions in low-HME non-responders (Figure S6D). Given these 1155 DA regions, we subtracted 324 regions that had the same outcome (“responder”) after ADE and ADE+BTZ treatment and those that did not respond (“non-responder”) with ADE+BTZ treatment (Appendix A). As a result, 401 chromatin regions were significantly more accessible in patients with high-HME profiles that responded to bortezomib-containing chemotherapy, while 430 open regions were found in patients with low-HME profiles who relapsed after bortezomib-containing chemotherapy. Among these, 65 and 74 regions, respectively, correlated with open chromatin corresponding to gene locations (Tables S3 and S4, respectively). To determine protein associations among these gene sets, we performed an SPIA pathway analysis that used the KEGG database, However, this revealed no dysregulated pathways based on overrepresentation and signaling perturbation accumulation. A String software analysis showed upregulations of WNT3, WNT5A and WNT6 in those with high HME levels who did not relapse after receiving ADE+BTZ, compared to patients with low-HME profiles who relapsed under this treatment regimen (Figure S6E). One component of the Wnt pathway that was analyzed in the RPPA is β-catenin. Upon Wnt stimulation, β-catenin phosphorylation is inhibited, which eventually leads to the transcription of the Wnt target genes. Corresponding with open chromatin regions for Wnt proteins, β-catenin phosphorylation on serine 33, 27 and threonine 41 was significantly lower in patients with the upregulation of HME in our cohort (*p* < 0.001, Figure S6F).

## 4. Discussion

This study on RPPA-based proteomics in pediatric AML shows that increased levels of the total and activated enzymes affecting histone and chromatin modification predict poor prognosis after conventional therapy, independent of the global histone methylation mark abundance. This is in line with a similar study on adult AML [20]. We demonstrated that this subgroup with high HME levels had more open chromatin regions surrounding promoter sites compared to those with lower expressions of HMEs and that these patients benefitted from bortezomib-containing chemotherapy in terms of overall survival. Notable was the unraveled open chromatin surrounding Wnt-signaling associated gene locations in those with high HME levels who responded to ADE+BTZ. Furthermore, we found that high HME levels inversely correlate with the FOXO3 protein expression. Patients with lower expressions of FOXO3 had adverse outcomes after ADE treatment, but this was ameliorated through the addition of bortezomib. Additionally, we report for the first time a FOXO3–vimentin correlation in AML.

For both adult and pediatric AML, the high-HME phenotype has been associated with poorer outcomes regardless of (cyto-)genetic risk stratification. Also, we show that a chromatin accessibility analysis revealed more open chromatin regions at protomer loci in the high-HME group compared to the low-HME group. Previous studies that integrated ATAC-seq and mRNA analysis demonstrated a positive correlation between gene promoter accessibility and transcribed genes [40,41]. The high-HME profile may therefore potentially reflect higher protein production. An impaired overactive proteome can lead to an overall increase in intracellular protein load and an increased accumulation of misfolded or aggregated proteins [42]. We believe that a dysfunctional proteasome might serve as a means to increase resistance to chemotherapy and decrease survival in high-HME patients. Dolfi et al. found that an increased protein synthesis rate positively correlates with relative protein content and is proportional to estimated metabolic fluxes and proliferation rates in an NCI-60 panel [43]. Cell metabolism differences have significant impact on the response to a variety of therapeutics, including antimetabolites (cytarabine) and topoisomerase II inhibitors (doxorubicin and etoposide) [43]. Cells with higher protein synthesis are significantly less sensitive to these chemotherapy agents than cells with lower synthesis rates and content. This is in agreement with our finding of poorer responses to ADE in high-HME profiles with more open and therefore more active chromatin.

We found that increased sensitivity to bortezomib was associated with higher histone and chromatin modifier expression levels. In this study, we provided several lines of support for this finding. First, patients with high HME levels treated with ADE had a 23% higher 3-year relapse risk compared with those with low HME levels and had more active chromatin, which could result in increased protein production. An increased protein load has been suggested as a predictor for enhanced sensitivity to proteasome inhibition [17,44,45]. A higher expression of HMEs itself has been linked to bortezomib-induced toxicity. For example, in multiple myeloma, bortezomib enhances apoptosis through targeting HDACs [12], predominantly through the downregulation of HDAC3 [46]. We argue that a similar mechanism of action is at work in AML, as we found that HDAC3 levels were significantly downregulated in responders to ADE+BTZ after 24 h of treatment, but not in the absence of bortezomib or in patients that were bortezomib resistant and relapsed (Figure S7).

Secondly, SIRT1 is another HME that is thought to have contributed to the outcomes in bortezomib-treated patients through FOXO3. SIRT1 promotes the deacetylation of FOXO3, leading to ubiquitination and finally FOXO3 proteasomal degradation. In this pediatric AML clinical trial, FOXO3 inactivation was independently associated with poor EFS and OS after ADE, an effect negated through additional BTZ treatment. Since SIRT1 results in the degradation of FOXO3, we conjecture that bortezomib inhibits FOXO3 degradation and, subsequently, would result in cell death [47,48]. We further speculate that lower FOXO3 levels are associated with reduced chemosensitivity against doxorubicin and etoposide. The apoptosis of leukemic cells after doxorubicin treatment has already been shown to be dependent upon FOXO3, and in neuroblastoma, FOXO3 sensitizes cells to apoptosis induced through a combination treatment of doxorubicin and etoposide [35,48]. Interestingly, we found no differences in viable cell counts after cytarabine therapy between cells cultured in vitro with or without FOXO3, but we did find increased resistance against doxorubicin and etoposide combination therapy in cells with FOXO3 knockdown, confirming our observation in RPPA-analyzed patient samples.

It is not surprising that total FOXO3 levels correlated negatively with vimentin protein and RNA, as vimentin is involved in sequestering and degrading FOXO3 [49]. Patients with low FOXO3 levels and relatively high vimentin levels (119/374 = 32%) had the largest benefit from the addition of bortezomib in terms of survival in the AAML1031 study. On the other hand, a small group (63/374 = 17%) of patients with high FOXO3 and vimentin levels trended toward worse survival with bortezomib. Vimentin is an intermediate filament that mediates cell integrity, migration and signaling and, when overexpressed in solid tumors, facilitates metastasis [37,49]. The role of vimentin is very versatile, and more research is needed to unravel its function in the presence of FOXO3 after proteasome inhibition. Remarkable is that by measuring FOXO3 and vimentin levels, we can identify 49% (32 + 17%) of newly diagnosed patients as those who might or might not receive bortezomib. The five-year overall survival improved 22%, in almost one third of the AML patients with low FOXO3 and high vimentin levels after the bortezomib addition.

Thirdly, we compared specific differentially accessible regions in high-HME responders to ADE+BTZ to low-HME non-responders and found that open regions were enriched for proteins that act in the Wnt pathway, including WNT3, WNT5A and WNT6. Aberrant activity of the Wnt signaling pathway has oncogenic properties, for example, mediating proliferation, migration and drug resistance, as shown in multiple myeloma. In the absence of Wnt proteins, β-catenin is phosphorylated on serine 33 and 37 and threonine 41, and then subsequently ubiquitinated, leading to its proteasomal degradation and thus low levels of cytoplasmic β-catenin. However, upon Wnt stimulation, β-catenin phosphorylation is inhibited; β-catenin accumulates and is translocated to the nucleus and activates Wnt target genes. Bortezomib can reduce the level of β-catenin and may inhibit cell proliferation and accelerate apoptosis that is caused by the Wnt target gene expression [50,51]. In agreement with open chromatin regions for Wnt proteins, β-catenin phosphorylation on serine 33 and 27 and threonine 41 was significantly lower in patients with an upregulation of HMEs in our cohort, suggesting that the baseline upregulation of the Wnt signaling pathway may sensitize cells to undergo apoptosis when treated with bortezomib.

On analyzing the ATAC-seq, instead of peak calling for the high read density, we used a window-based strategy to summarize read counts across the genome. The former identifies peaks representing accessible chromatin in each sample separately and thereby allows for qualitative comparisons between samples and conditions. However, one pitfall is that open regions identified via peak calling may not be of biological interest if the degree of accessibility does not change between samples. In contrast, the latter sliding window approach focuses on the quantitative variation in the accessible genome between conditions. This ‘differential accessibility’ approach focuses on sites of biological interest between samples.

## 5. Conclusions

In conclusion, the HME expression is variable among pediatric patients with AML. A high-HME profile is associated with low FOXO3 levels, more open chromatin, and a higher relapse risk prediction after ADE treatment. These patients might be the preferred candidates for the addition of bortezomib to standard chemotherapy. We speculate that enhanced proteasome inhibitor sensitivity in AML is related to a high HME expression that conveys more open chromatin and higher protein loads. Also, we suggest that high SIRT1 levels contribute to the proteasomal degradation of FOXO3 and that this effect is inhibited by bortezomib. As validated through in vitro experimentation, the knockdown of FOXO3 leads to higher leukemic cell counts and cells that are more resistant to doxorubicin and etoposide combination therapy. Thus, although bortezomib-containing chemotherapy did not improve outcomes in the entire population in the AAML1031 study, there is a group of patients that may benefit from bortezomib addition based on histone and chromatin modification. This study provides insights into the mechanisms linked to bortezomib sensitivity in pediatric AML.

## Figures and Tables

**Table 1:** Patient demographics and clinical characteristics by matrix cluster membership.

Patients (*n* = 410)		% of Patients	C1	C2	C3	C4	*p* Value
Total		100%	29%	17%	26%	28%	-
Sex	Female	49%	50%	47%	50%	48%	0.97
Age (years)	<1	12%	16%	14%	7%	12%	0.48
	2 till 10	33%	33%	31%	37%	32%	
	>11	54%	51%	54%	56%	56%	
Race	Black	12%	13%	8%	10%	15%	0.58
Ethnicity	Hispanic	17%	18%	19%	16%	16%	0.93
CNS status	1	60%	58%	64%	65%	54%	0.74
	2	30%	31%	27%	26%	35%	
	3	10%	11%	9%	9%	11%	
WBC	>100,000	25%	24%	24%	25%	26%	0.97
Cytogenetics	Inv(16)/t(16;16)	14%	19%	20%	9%	9%	0.02
	t(8;21)	16%	14%	17%	11%	21%	0.23
	Normal	28%	24%	24%	31%	31%	0.55
	t(9;11)(p22;q23)/11q23	18%	18%	17%	21%	14%	0.55
	Monosomy −5, −7, or +8	8%	7%	6%	14%	4%	0.04
	Other	17%	18%	16%	13%	21%	0.46
FLT3-ITD	Mutant	22%	19%	16%	30%	23%	0.12
	High allelic ratio (>0.4)	15%	10%	10%	24%	13%	0.01
CEBPA	Mutant	10%	4%	10%	7%	17%	0.01
NPM1	Mutant	11%	13%	10%	9%	12%	0.84
Treatment	ADE	40%	45%	40%	34%	41%	0.26
	ADE+BTZ	51%	49%	56%	52%	50%	
	ADE+SFB ^††^	9%	7%	4%	14%	9%	
Risk stratification ^†^	High risk	30%	28%	20%	41%	27%	0.02
Complete	ADE patients (*n* = 164)	85.4%	88.7%	82.1%	83.3%	85.1%	0.84
Remission	ADE+BTZ patients (*n* = 210)	84.8%	79.3%	79.5%	89.3%	89.5%	0.26
Relapse	ADE patients (*n* = 140)	34.8%	26.4%	25.0%	41.7%	44.7%	0.13
	ADE+BTZ patients (*n* = 178)	34.3%	32.8%	28.2%	37.5%	36.8%	0.77

^†^ AAML1031 protocol risk group definition: low risk: inv(16)/t(16;16) or t(8;21), or NPM or CEBPα mutation; high risk: FLT3/ITD+ with high allelic ratio ≥ 0.4, or monosomy 5/del5q or 7, without low-risk features; risk classification was missing for 10 patients. ^††^ SFB = Sorafenib.

**Table 2:** Univariable analysis in patients treated with ADE.

Univariable		RR from End of Course 2				OS from Study Entry			
		N	HR	95%CI	*p* Value	N	HR	95%CI	*p* Value
Matrix	C1	47	1			53	1		
	C2	23	1.04	0.43–2.54	0.928	28	1.01	0.40–2.52	0.990
	C3	30	2.17	1.04–4.51	0.038	36	1.80	0.84–3.82	0.129
	C4	40	2.14	1.11–4.13	0.023	47	1.88	0.94–3.75	0.075
HME	High	70	1			83	1		
	Low	70	0.47	0.28–0.80	0.005	81	0.54	0.31–0.94	0.030
HMM	High	63	1			75	1		
	Low	77	0.819	0.49–1.37	0.449	89	0.84	0.50–1.43	0.524

**Table 3:** Multivariable competing risk regression analysis in patients treated with ADE.

Multivariable		RR from End of Course 2				OS from Study Entry			
		N	HR	95%CI	*p* Value	N	HR	95%CI	*p* Value
HME	High	70	1			82	1		
	Low	70	0.45	0.26–0.77	0.004	79	0.59	0.33–1.06	0.077
Age (year olds)	<1	15	2.44	1.15–5.16	0.020	19	2.70	1.20–6.08	0.017
	2–10	47	1			51	1		
	>11	78	0.86	0.47–1.58	0.623	91	1.24	0.65–2.35	0.520
AAMl1031	Low risk	119	1			126	1		
risk group definition	High risk	21	0.79	0.35–1.79	0.577	35	2.96	1.66–5.27	<0.001

## Data Availability

The raw data supporting the conclusions of this article will be made available by the authors on request.

## Supplementary Materials

The following supporting information can be downloaded at: https://www.mdpi.com/article/10.3390/cancers16081448/s1, Figure S1: Fragment size distribution of ATAC-seq reads per sample; Figure S2: Lower total FOXO3 protein expression in the high-HME cluster compared to the low-HME cluster (*p* = 2.4 × 10^−6^); Figure S3: Viable cell counts in shFOXO3 (blue) and shGIPZ (red) OCI-AML3 cell lines treated with 1, 3 or 10 nM of bortezomib after 72 h; Figure S4: (A) Volcano plot showing differentially expressed genes between low- and high-FOXO3 expressors. Each dot represents a gene with genes lower than a −1 log2-fold change (FC) shown in blue, and genes that were higher than 1 log2-FC, in red. Others are shown in light grey. A total of 4685 genes were significantly differentially expressed. (B,C) Pathway topology analysis (SPIA) revealed that among the 137 pathways in the KEGG database, *Focal adhesion* and *ECM-receptor interaction* were significantly dysregulated based on over-representation and signaling perturbation accumulation; Figure S5: (A) Waterfall plot showing proteins that significantly correlated with FOXO3 on the RPPA based on Pearson’s correlation coefficient > 0.25. Vimentin (VIM, red-lined) was the protein that showed the strongest negative correlation with FOXO3 on the array. (B) Scatterplot showing the negative correlation between vimentin (*y*-axis) and FOXO3 (*x*-axis), r = −0.47, *p* < 2.2 × 10^−16^); (C) overall and (D) event-free survival of patients treated with ADE (*n* = 164) stratified by FOXO3 and vimentin protein levels; (E) overall survival in patients with low FOXO3 and high VIM expressions according to treatment regime (ADE vs. ADE+BTZ); (F) overall survival in patients with high FOXO3 and high VIM expressions according to treatment regime (ADE vs. ADE+BTZ); Figure S6: (A) Mean accessibility of each region (*x*-axis) and variances (*y*-axis) plotted as log2 counts per million (CPM). Variances are re-scaled to quarter-root variances (“standard deviations”) and plotted against the mean accessibility of each region. Trend is shown in red; (B) log2 residual standard deviations against mean log-CPM values. The average log2 residual standard deviation is marked by a horizontal blue line. In both (A) and (B), each dot represents a region; (C) mean-difference plot with summary data of log-fold changes (FCs) vs. log-CPM values. Red dots represent regions that were more accessible in high-HME patient samples that responded to ADE+BTZ compared to those with low-HME samples and that relapsed after ADE+BTZ treatment. Blue dots represent the regions that were less accessible between those groups. (D) Venn diagram showing uniquely and overlapping differential accessible chromatin regions of patients with high HME levels that responded to ADE+BTZ therapy (red). HIGH = High-HME; LOW = Low-HME; ADE = ADE treatment; ADEB = ADE+BTZ treatment; RESP = No relapse; NONRESP = Relapsed patients; (E) String software analysis of gene locations that were significantly more accessible in those with high HME levels who did not relapse after receiving ADE+BTZ, compared to patients with low-HME profiles who relapsed under this treatment regime. Proteins that play a role in the Wnt pathway are shown in red (WNT3 WNT5A and WNT6); (F) Beta-catenin phosphorylation on serine 33 and 37 and threonine 41 in the high-HME cluster compared to the low-HME cluster (*p* = 2.6 × 10^−8^); Figure S7: (A) *p*-values for between 0 and 24 h of the protein expressions of all HMEs individually divided by received treatment (ADE or ADE+BTZ) and response (no relapse or relapse after two courses of induction therapy). (B) HDAC3 and (C) HDAC6 levels decreased after bortezomib-containing chemotherapy (ADE+BTZ) 24 h after chemotherapy exposure in patients who experienced no relapse after two courses of induction therapy (*p* = 0.0011 and *p* = 0.025, respectively), but not after ADE therapy, or when relapse occurred. Figure S8: (A) uncropped Western blot of FOXO3A; (B) p53; (C) BIM and (D) tubulin. Lanes from right to left on every blot are OCI-AML3 shGIPZ, OCI-AML shFOXO3, THP1 shGIPZ and THP1 shFOXO3; (E) net protein, background densities and intensity ratios; (F) graphical presentations of the net protein/loading control ratio. Table S1: A total of 171 genomic regions were significantly more accessible in all high-HME expressors compared to low-HME expressors; Table S2: Univariate and multivariate cox regression analysis for event-free survival (EFS) of all included patients based on FOXO3 expression (*n* = 410); Table S3: A total of 65 regions correlated with open chromatin corresponding to gene locations in patients with high-HME profiles that responded to bortezomib-containing chemotherapy; Table S4: A total of 74 regions correlated with open chromatin corresponding to gene locations in patients with low-HME profiles who relapsed after bortezomib-containing chemotherapy.

## Appendix A

### Appendix A.1. Additional Information

#### Appendix A.1.1. Patient Population

Outcome data were available for 410 of the 483 patients and were frozen on 30 June 2019. By the end of course 2, 348 (84.9%) of 410 patients were in complete remission (CR) while 31 (7.6%) were refractory or had died before response assessment. Relapse occurred in 138 patients (34%), and at the end of follow-up, 286 patients were still alive (70%). Of the patient samples with available clinical data, 164 patients were treated with standard ADE, and 210 patients received additional bortezomib (ADE+BTZ). Thirty-six patients with a high allelic ratio (HAR) FLT3-ITD (>0.4) received additional sorafenib (ADE+SFB) [31]. The research aim of this trial was to evaluate the additional outcome effect of BTZ added to ADE; therefore, patients with ADE+SFB were excluded from the efficacy analysis in this study.

#### Appendix A.1.2. Sample Processing

After collection, samples were placed under refrigeration until shipment to the processing site at Baylor College of Medicine. Samples were shipped via overnight courier with at least one cold pack. Samples were assessed for viability upon arrival and were processed if viability was >80%. Samples were enriched for leukemic cells via sucrose gradient separation, followed by CD3/CD19 depletion via Magnetic Antibody Conjugated Sorting (AutoMACS, Miltenyi Biotec) if the blast percentage was <85%. Samples were then normalized to a concentration of 10,000 cells/µL, and whole-cell lysates were prepared as previously described [19,20,21,22,23].

#### Appendix A.1.3. RPPA Methodology

The proteomic profiling of the AML patient samples was generated using RPPAs performed as previously described [4,19,20,21,22,23]. Patient sample lysates were printed in five serial dilutions onto slides along with normalization and expression controls. Protein expression levels were normalized relative to the mean expression of non-malignant bone marrow CD34+ cells from 30 healthy donor samples (20 pediatric and 10 adult samples). Slides were probed with 296 strictly validated primary antibodies, a secondary antibody to amplify the signal and a stable dye for signal detection. We simultaneously analyzed the expression of the H3 core protein with 5 histone modification methylation marks (H3K4me2, H3K4me3, H3K9me2, H3K27me3, H3K36me3) and 16 histone and/or chromatin modifying enzymes (ASH2L, BMI1, BRD4, NCL, CLPP, HDAC1, HDAC2, HDAC3, HDAC6, hnRNPK, JMJD6, KDM1A, NPM1, SIRT1, SIRT6 and WTAP). Stained slides were analyzed using Microvigene^®^ software (Version 3.4, Vigene Tech, Carlisle, MA, USA) to determine protein concentrations.

#### Appendix A.1.4. RPPA Quality Control

To exclude low-quality samples analyzed using the RPPA, we removed samples that showed a greater number of proteins with extreme levels of expression, using a cut-off value of a +3SD mean protein expression. Initially, 1524 samples were analyzed, of which 1.4% (*n* = 22) were excluded as they did not meet this quality control standard [52].

#### Appendix A.1.5. shRNA Knockdown Cells

Briefly, we generated lentivirus through the transient transfection of HEK293T cells with equimolar mixtures of transfer vector and packaging plasmids pMD2.6 and psPAX2 (kind gifts from Didier Trono, Addgene plasmids 12,259 and 12,260, respectively), and lentiviral supernatants were clarified via centrifugation (300× *g*), and then passed through 0.45 micron SFCA filters before use. Infected cells were selected with puromycin, and knockdown was verified using a Western blot analysis. We obtained the best knockdown in both cell lines with transfer vector V3LHS_641765, which delivered the following mature antisense sequence: GCAGTGACAGGTTGTGCCG.

#### Appendix A.1.6. Cell Treatment and Cytotoxicity

Cells were treated with various doses of Bortezomib (Bio-connect) and/or etoposide and/or doxorubicin and/or ABT-199 (Selleck Chemicals, Houston, TX, USA) various times. Cell death was assessed via trypan blue staining. To determine that the mechanism of cell death was apoptosis, cells were stained with Annexin V (Roche Diagnostics, Indianapolis, IN, USA)/4′,6-diamidino-2-phenylindole (DAPI), and the percentages of apoptotic cells were assessed via flow cytometry. Apoptotic cells were identified as positive for Annexin V staining using a Becton Dickinson LSR II flow cytometer (Becton Dickinson, San Jose, CA, USA). Total viable cells were determined using total Annexin V negative/DAPI negative cells with counting beads (Roche) included to determine the total cell number.

#### Appendix A.1.7. ATAC Library Preparation and Sequencing

Viable cryopreserved peripheral blood samples containing >90% leukemic blasts from 24 patients were thawed and washed twice with ice-cold PBS via spinning at 300× *g* for 5 min. Ficoll density gradient centrifugation was performed to separate viable cells from dead cells following the manufacturer’s protocol. Sequencing libraries were prepared as previously described by Corces et al. (2017) [53]. In short, 50,000 viable cells were selected per sample and treated with 50 μL ATAC resuspension buffer containing 0.1% NP40, 0.1% Tween-20 and 0.01% Digitonin for cell lysis. Samples were incubated on ice for 3 min, and nuclei were collected after centrifugation for 10 min at 4 °C. The nuclei were then incubated with 25 μL transposition mixture containing 2 μL Tn5 transposase (Illumina, San Diego, CA, USA), 12.5 μL 2× TD buffer and nuclease-free water for 30 min at 37 °C. The reaction was cleaned using the MinElute kit (Qiagen, Hilden, Germany), amplified during 12 cycles in a thermocycler according to the described thermal profile with the Illumina index primers, and the final PCR product was purified using the aforementioned Qiagen kit. Libraries were then size-selected on gel to exclude small fragments smaller than 50 bp. The library quality was analyzed using the DNA tape station kit (Agilent technologies, Santa Clara, CA, USA), and its quantity, using the Qubit fluorometer (LifeTechnologies, Paisley, UK). Finally, libraries were sequenced on the Illumina HiSeq2500 platform with 40 bp paired-end sequencing. ATAC-seq libraries were sequenced to an average of 32.5 million reads per sample, resulting in a total of 780 million sequencing reads.

#### Appendix A.1.8. ATAC-seq Data Analysis

Pre-alignment quality control was performed using FastQC, which was followed by adapter trimming and quality filtering from the raw fastq files using fastp v0.20.1 [54]. Trimmed reads were mapped per sample to the reference human genome CRCh38.p38 using Bowtie2 v2.4.1 with *very-sensitive-X 2000* settings [55]. Picard *MarkDuplicates* was then applied to remove PCR duplicates [56]. Sorting and ‘bamtobed’ conversion were performed using SAMtools (version 1.12) [57]. Bash scripts were used to adjust for Tn5 cut sites (5 bp forward and 4 bp reverse) and for converting to a minimal BEDPE format as described by Reske et al. [58]. For a differential accessibility analysis between low- and high-HME samples, non-linear loess normalization was performed before windows were clustered into regions using the R package *csaw* (version 1.16.1) [59]. Genomic enriched windows that were less than 100 bp apart were considered to be adjacent and were grouped into the same cluster (‘region’). ChIPseeker was used for the annotation of genomic regions, for the visualization of distance to transcription start sites (TSS) and for estimating the significance of overlap among the data sets (low-HME vs. high-HME) [60]. The sequencing track figures were generated in R using the Gviz package (4.3) [61].

#### Appendix A.1.9. Characterization of the Open Chromatin Signature for High-HME Patients That Responded to Bortezomib-Containing Chemotherapy

We used *csaw* to count the extended reads of each sample that overlapped a sliding window at spaced (50 bp) positions across the genome (maximum fragment length = 1000) with a minimum mapping quality score for aligned reads of 30. The minimum count sum across libraries for each window was set as 10 (default). Reads mapping to mitochondrial DNA were excluded as well as unpaired alignments. Windows were filtered using the threshold of a 2-fold increase in enrichment over a 2 kb neighborhood abundance of each window (local enrichment filtering). The raw library sizes were then normalized using the TMM (trimmed mean of M values) method, which calculates the effective library size, and the sizes were then used as part of the per-sample normalization. The TMM method adjusts library sizes based on the assumption that most regions are not DA. Count data were transformed into log2 counts per million (logCPM) using the limma-voom function [38,62]. The mean–variance relationship among samples was estimated to compute the final observation-level weights. Before DA analysis, batch effects and other redundant variations in the experiment were removed using the sva package. First, we defined the variable of interest as the combined status of the sample (HME status, treatment and response; thus, for example: LOW-ADE-RESP) and the adjustment variables as the HME status (LOW vs. HIGH), treatment (ADE vs. ADE+BTZ) and response (responder vs. non-responder). We ran sva and identified 2 ‘surrogate variables’ and then included these in the design matrix. We were mostly interested in differential accessible regions between high-HME classified patients that responded to the ADE+BTZ treatment compared to low-HME patients that relapsed after the same treatment. We formulated this comparison as HIGH-ADEB-RESP vs. LOW-ADEB-NONRESP. DA regions between the following contrasts were then removed from the main comparison.

- DA regions that are associated with the same outcome regardless of the treatment include the following:
  ○ HIGH-ADEB-RESP vs. HIGH-ADE-RESP.
- DA regions that are associated with the same treatment but with different outcomes include the following:
  ○ HIGH-ADEB-RESP vs. HIGH-ADEB-NONRESP;
  ○ LOW-ADEB-NONRESP vs. LOW-ADEB-RESP.

Finally, we fitted the linear model to the data and tested for DA using limma and a cut-off of *p* < 0.01.
