# Supplementary material for: Chromatin Profiles Are Prognostic of Clinical Response to Bortezomib-Containing Chemotherapy in Pediatric Acute Myeloid Leukemia: Results from the COG AAML1031 Trial
Source: Cancers (Basel). 2024 Apr 9;16(8):1448. doi: 10.3390/cancers16081448 (PMC11048007; doi:10.3390/cancers16081448)

A

		No Relapse	Relapse after EO12
ASH2L	ADE	0.000	0.031
	ADE+BTZ	0.000	0.013
BMI1	ADE	0.790	0.570
	ADE+BTZ	0.690	0.670
BRD4	ADE	0.011	0.190
	ADE+BTZ	0.082	0.110
CLPP	ADE	0.040	0.460
	ADE+BTZ	0.510	0.300
hnRNPK	ADE	0.001	0.190
	ADE+BTZ	0.001	0.018
JMJD6	ADE	0.007	0.100
	ADE+BTZ	0.003	0.010
KDM1A	ADE	0.000	0.027
	ADE+BTZ	0.000	0.001
NCL	ADE	0.000	0.064
	ADE+BTZ	0.000	0.003
NPM1	ADE	0.180	0.390
	ADE+BTZ	0.031	0.033
HDAC1	ADE	0.700	0.590
	ADE+BTZ	0.800	0.990
HDAC2	ADE	0.000	0.100
	ADE+BTZ	0.003	0.130
HDAC3	ADE	0.100	0.220
	ADE+BTZ	0.001	0.210
HDAC6	ADE	0.120	0.610
	ADE+BTZ	0.025	0.110
SIRT1	ADE	0.021	0.014
	ADE+BTZ	0.037	0.980
SIRT6	ADE	0.100	0.230
	ADE+BTZ	0.090	0.420
WTAP	ADE	0.000	0.076
	ADE+BTZ	0.000	0.002
H3K4Me2	ADE	0.240	0.360
	ADE+BTZ	0.096	0.620
H3K4Me3	ADE	0.006	0.130
	ADE+BTZ	0.003	0.230
H3K9Me2	ADE	0.150	1.000
	ADE+BTZ	0.091	0.670
H3K27Me3	ADE	0.130	0.760
	ADE+BTZ	0.110	0.300
H3K36Me3	ADE	0.002	0.220
	ADE+BTZ	0.015	0.028

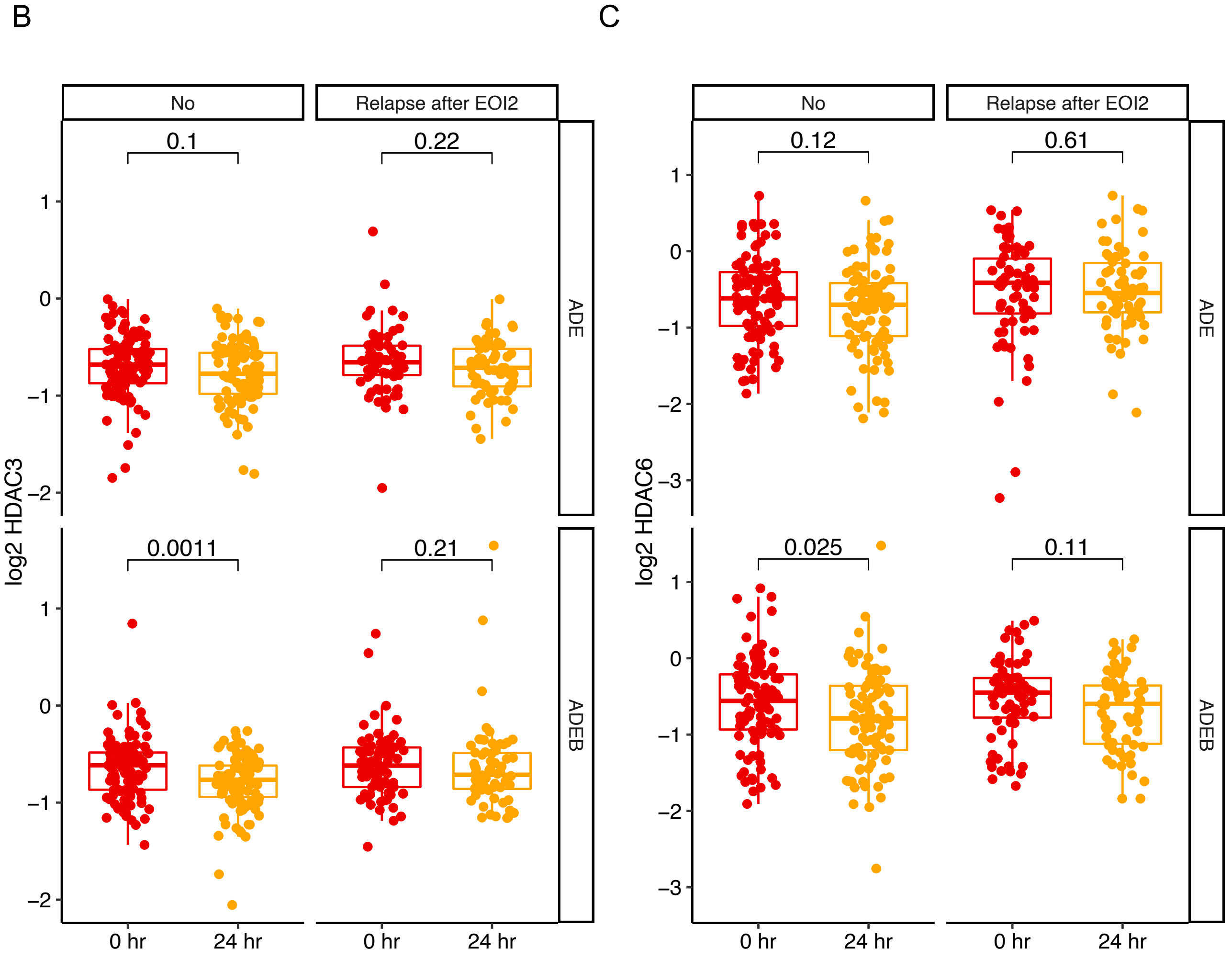

Supplement: Supplementary file 1 [file cancers-16-01448-s001.zip › Supplementary Figure S7.pdf]
