# Supplementary material for: Chromatin Profiles Are Prognostic of Clinical Response to Bortezomib-Containing Chemotherapy in Pediatric Acute Myeloid Leukemia: Results from the COG AAML1031 Trial
Source: Cancers (Basel). 2024 Apr 9;16(8):1448. doi: 10.3390/cancers16081448 (PMC11048007; doi:10.3390/cancers16081448)

A FOXO3A

B p53

C BIM

D tubulin

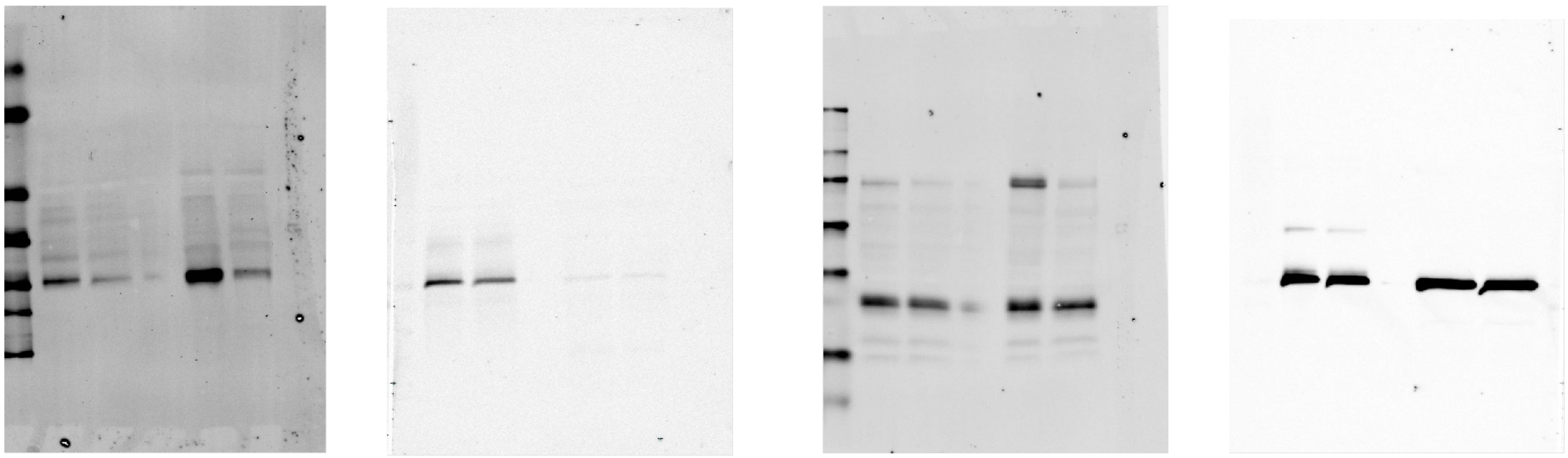

E

	FOXO3A				p53				BIM				tubulin (loading control)			
	OCI-AML3		THP.1		OCI-AML3		THP.1		OCI-AML3		THP.1		OCI-AML3		THP.1	
	shGIPZ	shFOXO3	shGIPZ	shFOXO3	shGIPZ	shFOXO3	shGIPZ	shFOXO3	shGIPZ	shFOXO3	shGIPZ	shFOXO3	shGIPZ	shFOXO3	shGIPZ	shFOXO3
Protein	150	117	202	131	98	80	22	23	142	129	139	142	91	92	126	119
Background	94	93	98	94	21	22	18	18	57	55	55	53	6	7	7	8
Net protein	56	24	104	37	77	58	4	5	85	74	84	89	85	85	119	111
Ratio	0,659	0,282	0,874	0,333	0,906	0,682	0,034	0,045	1,000	0,871	0,706	0,802	1	1	1	1

F

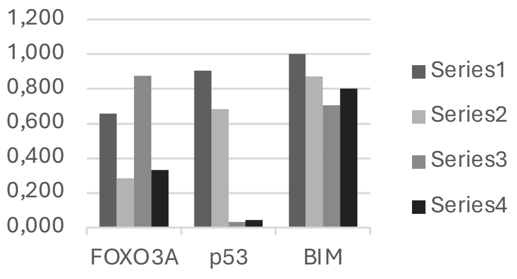

Supplement: Supplementary file 1 [file cancers-16-01448-s001.zip › Supplementary Figure S8.pdf]
