# Supplementary figures and images for: Chromatin Profiles Are Prognostic of Clinical Response to Bortezomib-Containing Chemotherapy in Pediatric Acute Myeloid Leukemia: Results from the COG AAML1031 Trial
Source: Cancers (Basel). 2024 Apr 9;16(8):1448. doi: 10.3390/cancers16081448 (PMC11048007; doi:10.3390/cancers16081448)

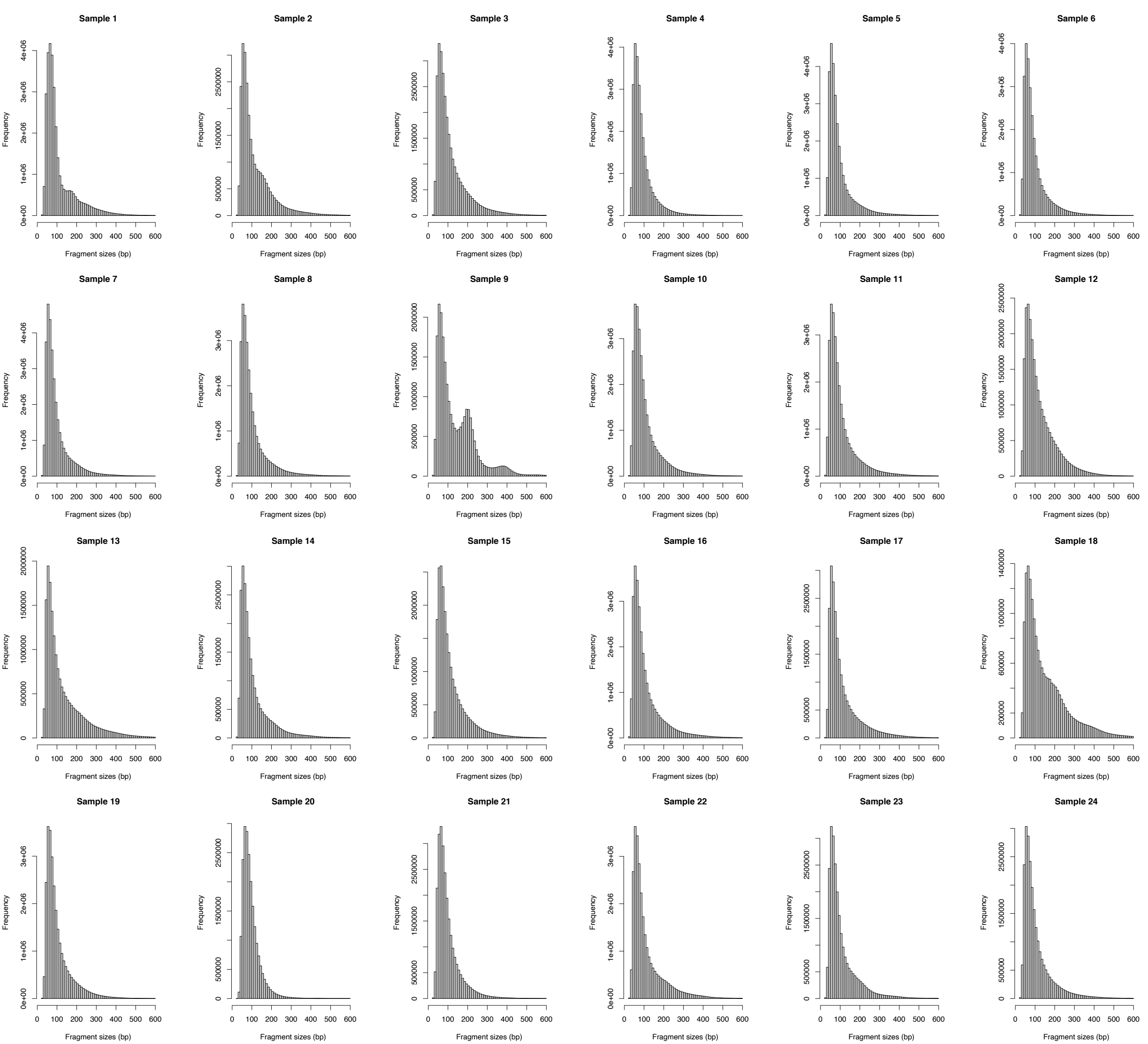

Supplement: Supplementary file 1 [file cancers-16-01448-s001.zip › Supplementary Figure S1.pdf]

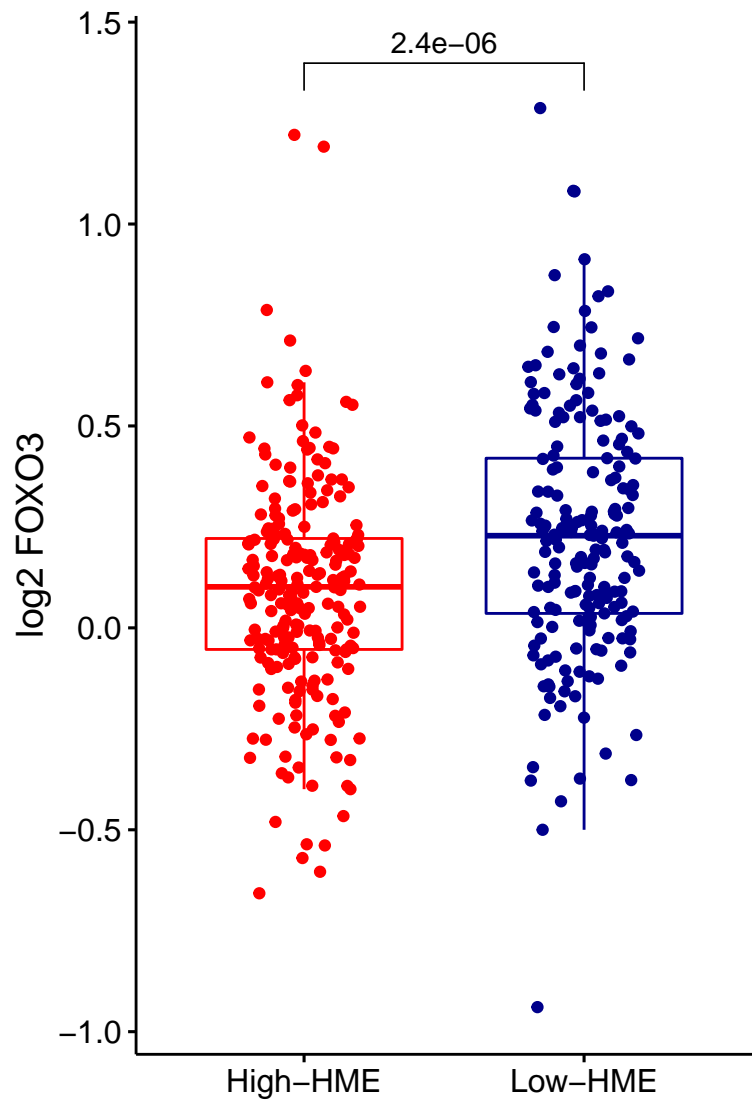

Supplement: Supplementary file 1 [file cancers-16-01448-s001.zip › Supplementary Figure S2.pdf]

# OCI-AML3

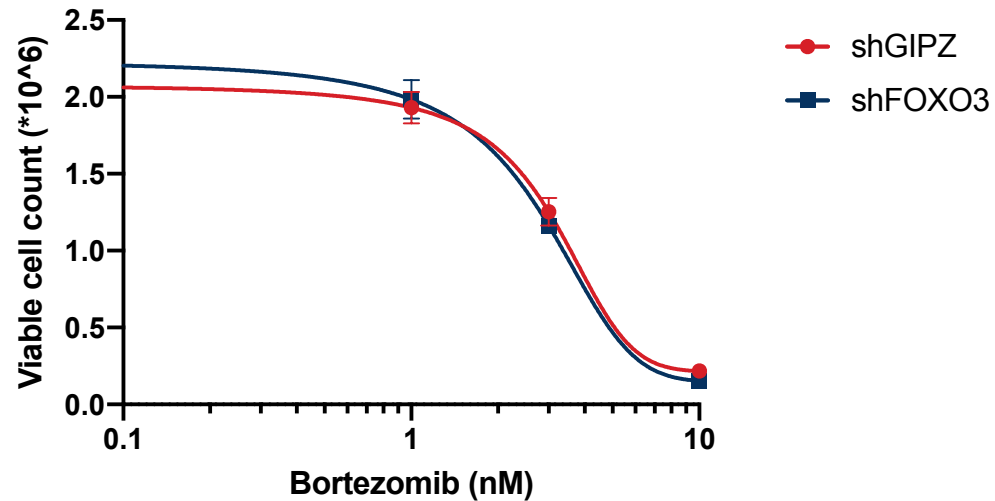

Supplement: Supplementary file 1 [file cancers-16-01448-s001.zip › Supplementary Figure S3.pdf]

### Differentially expressed genes between low and high FOXO3 expressors

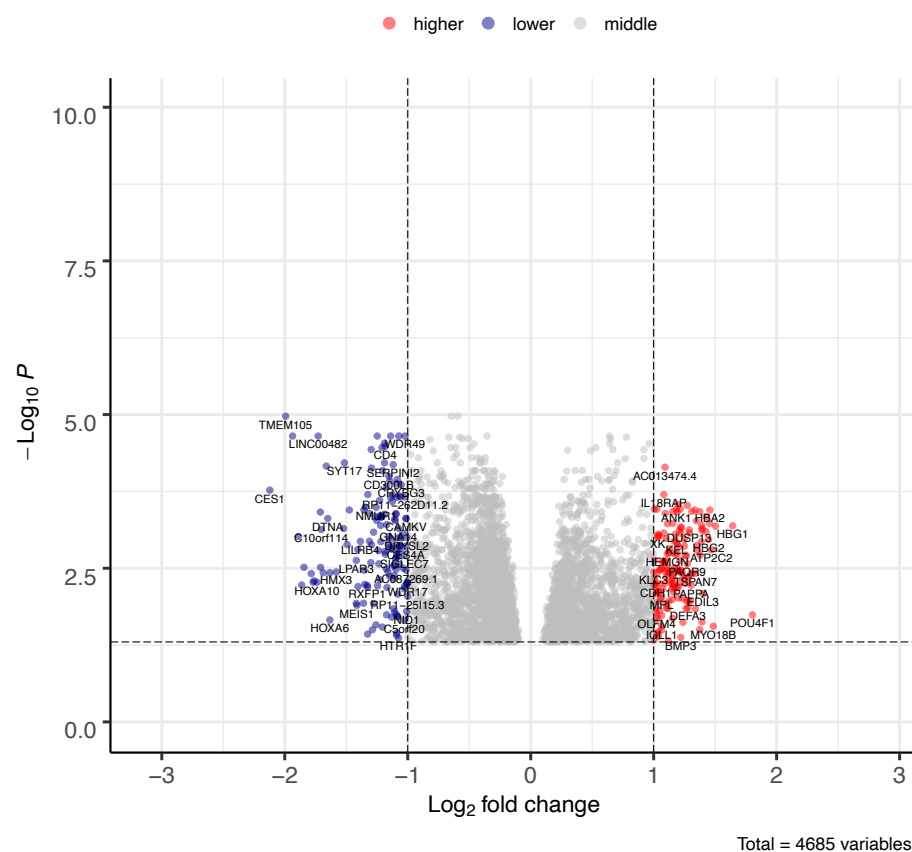

# B

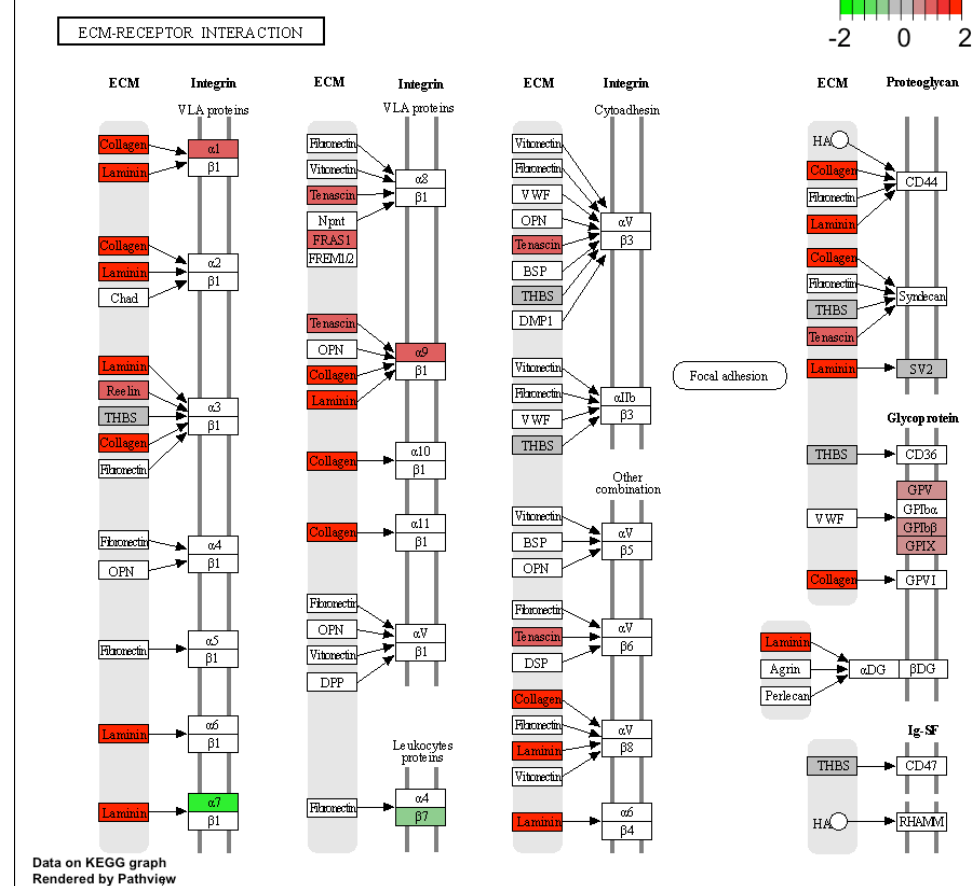

C

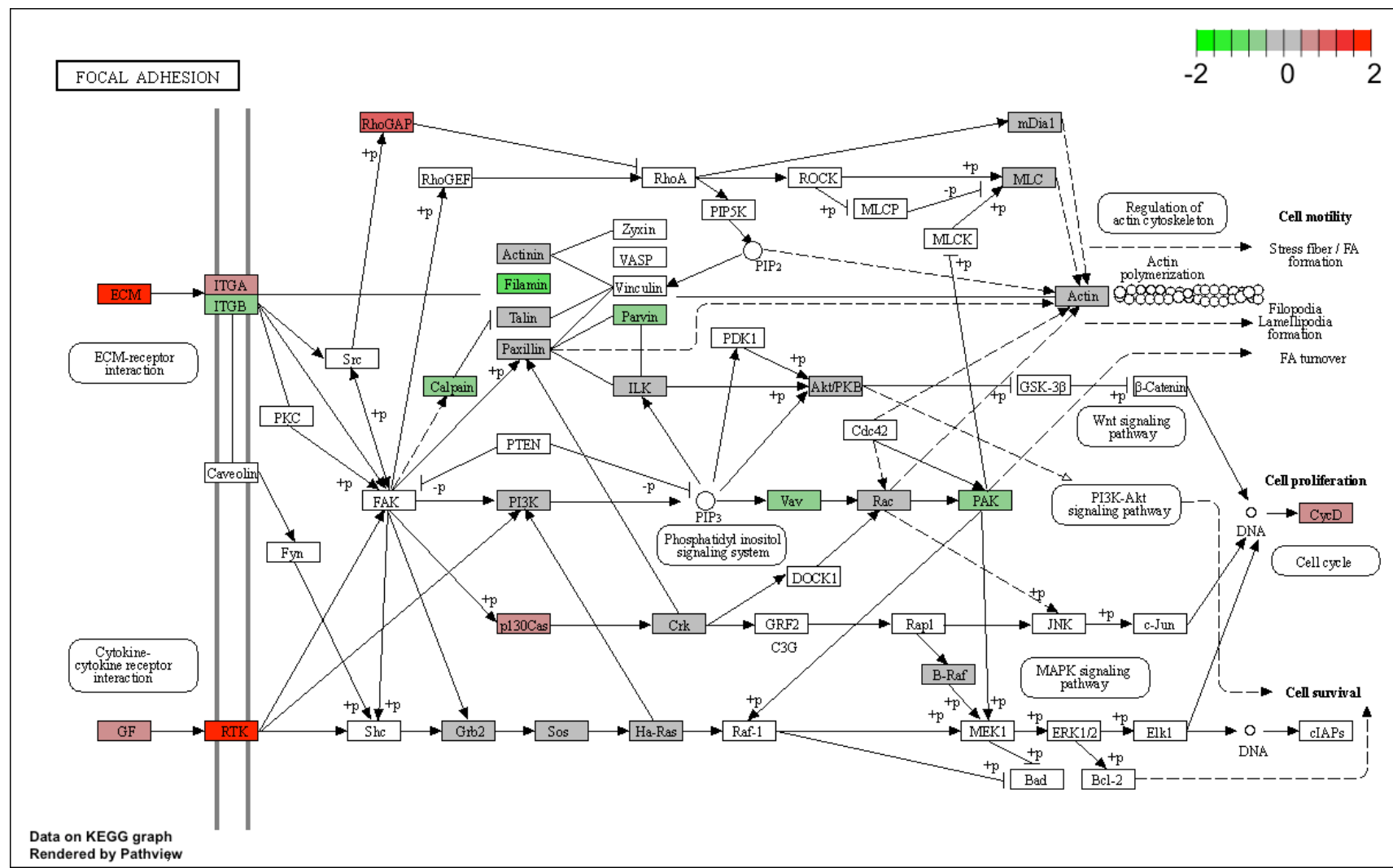

Supplement: Supplementary file 1 [file cancers-16-01448-s001.zip › Supplementary Figure S4.pdf]

A

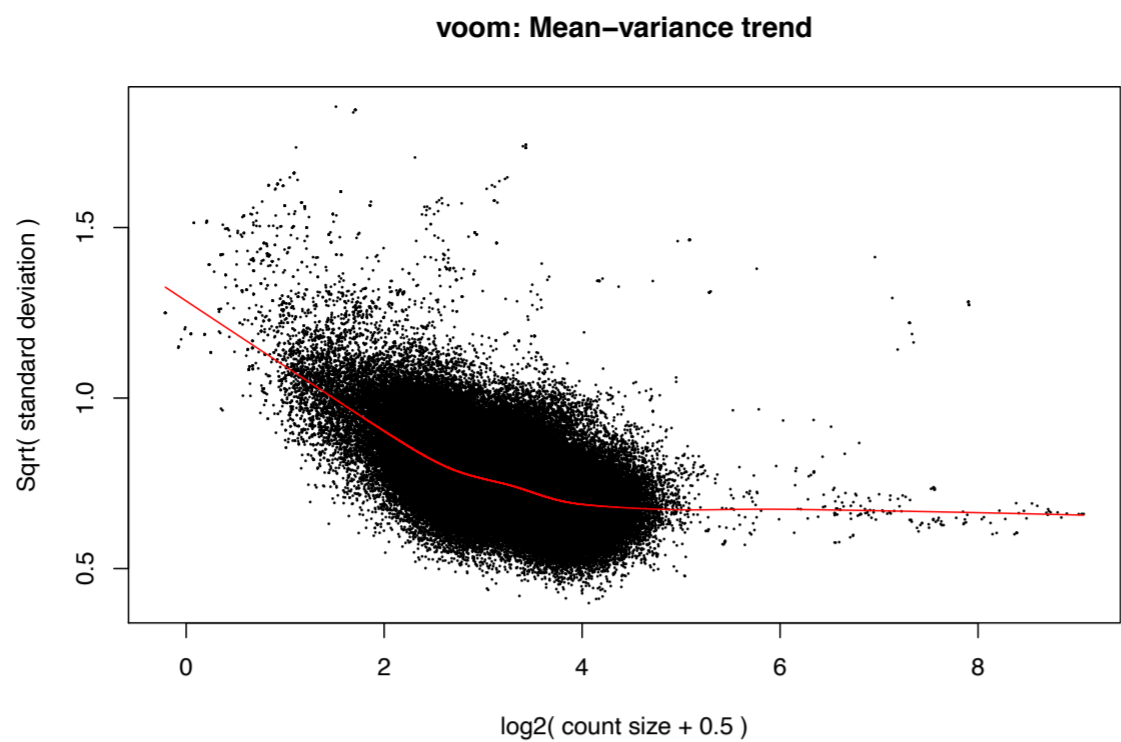

B

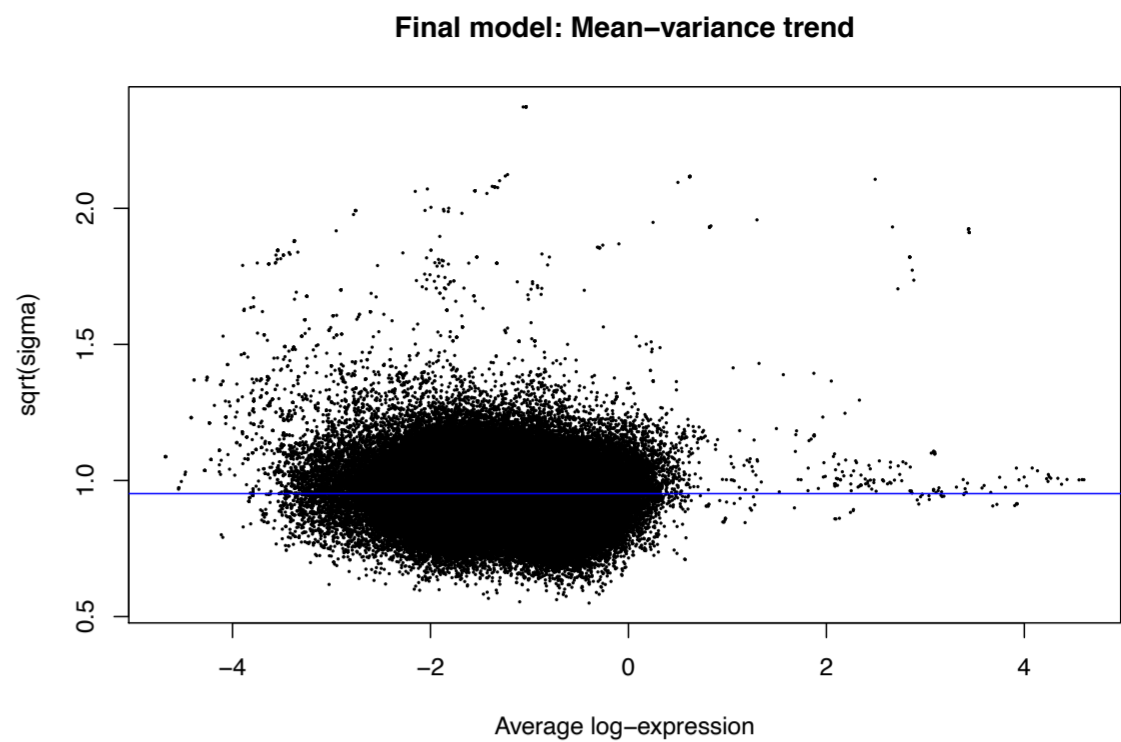

C

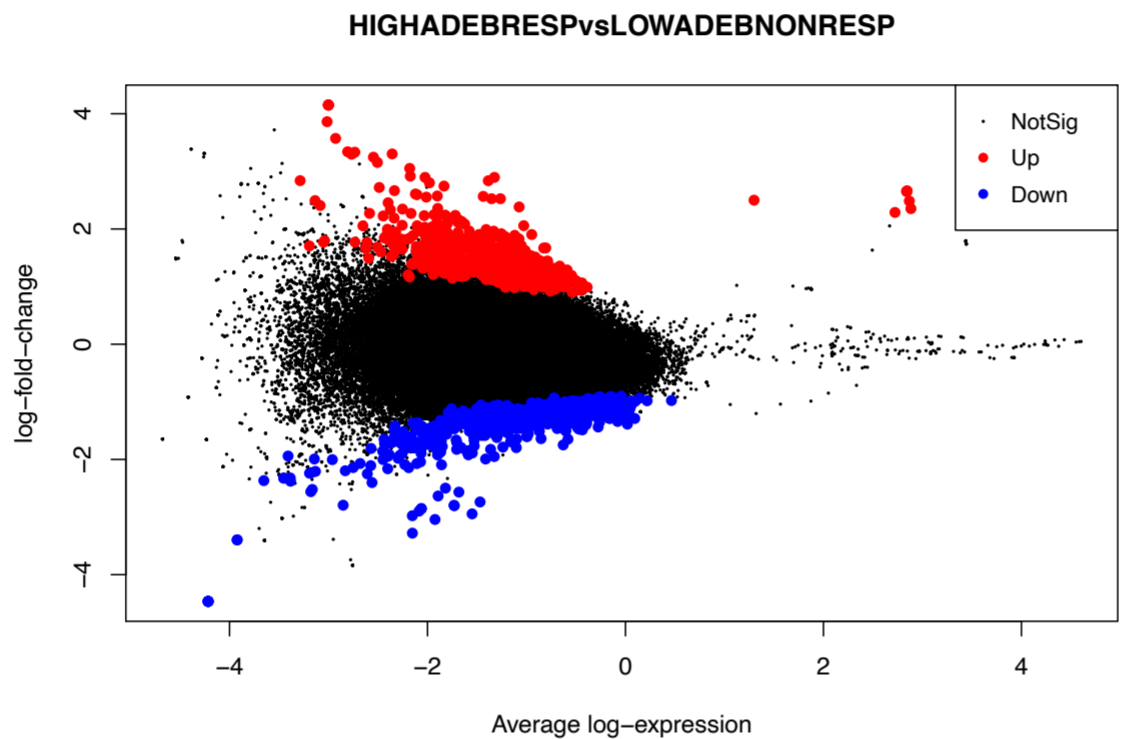

D

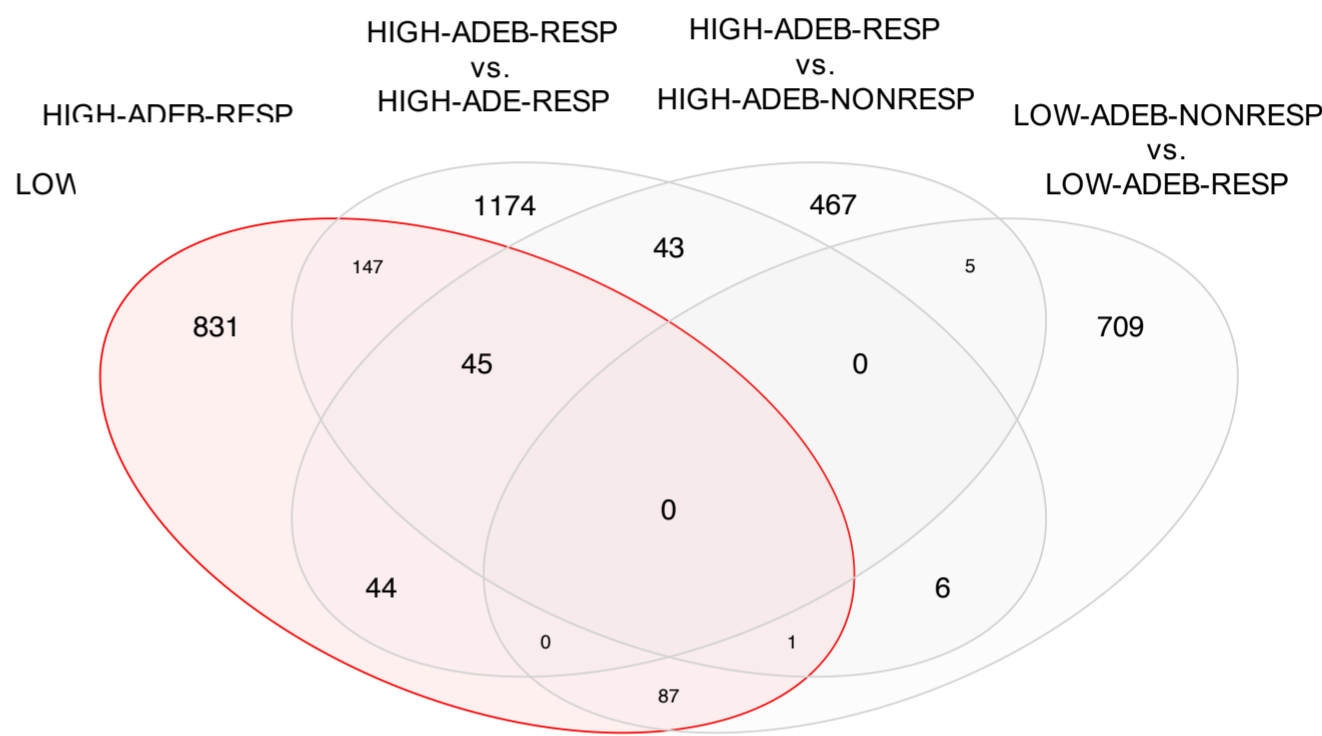

E

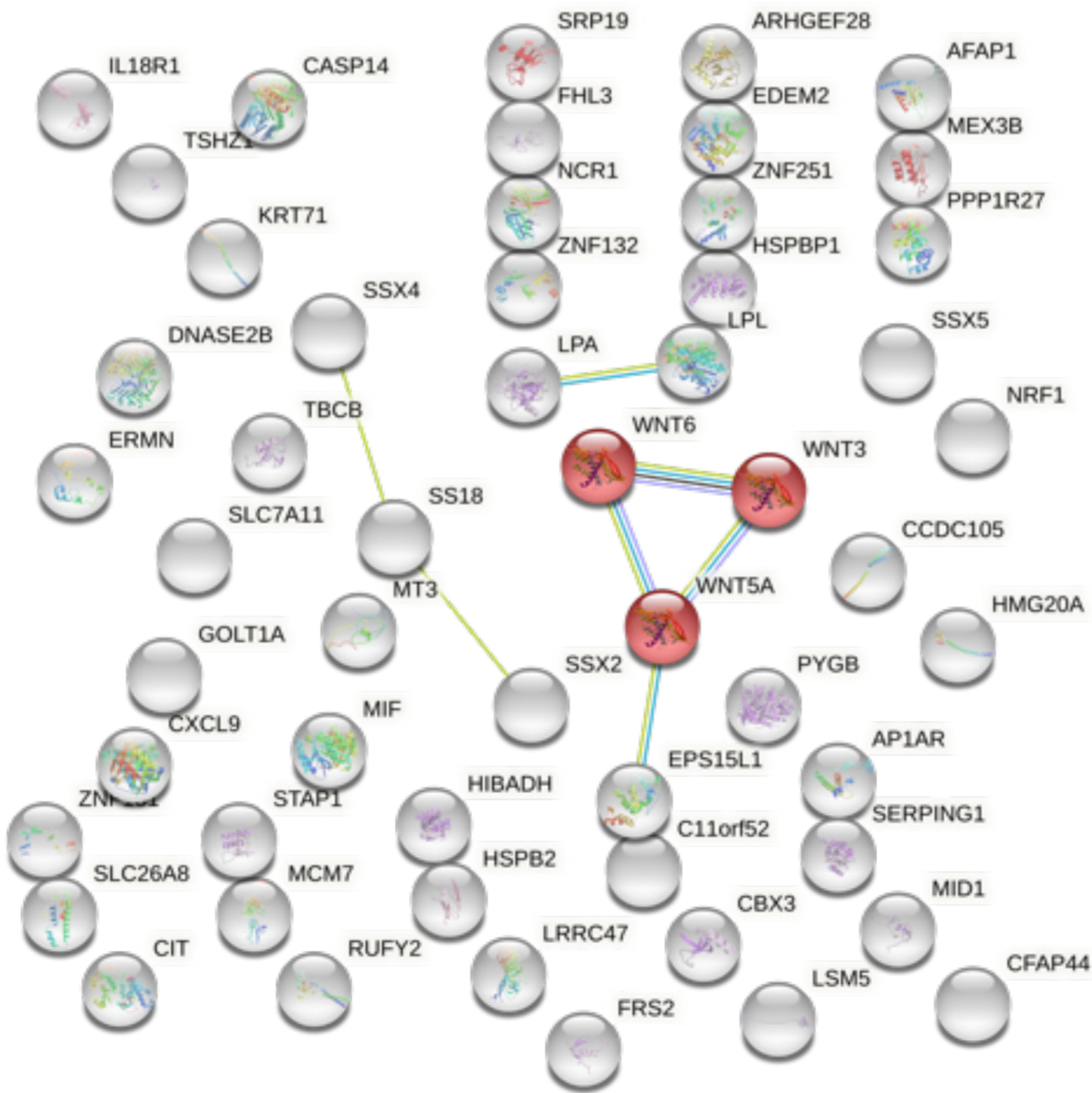

F

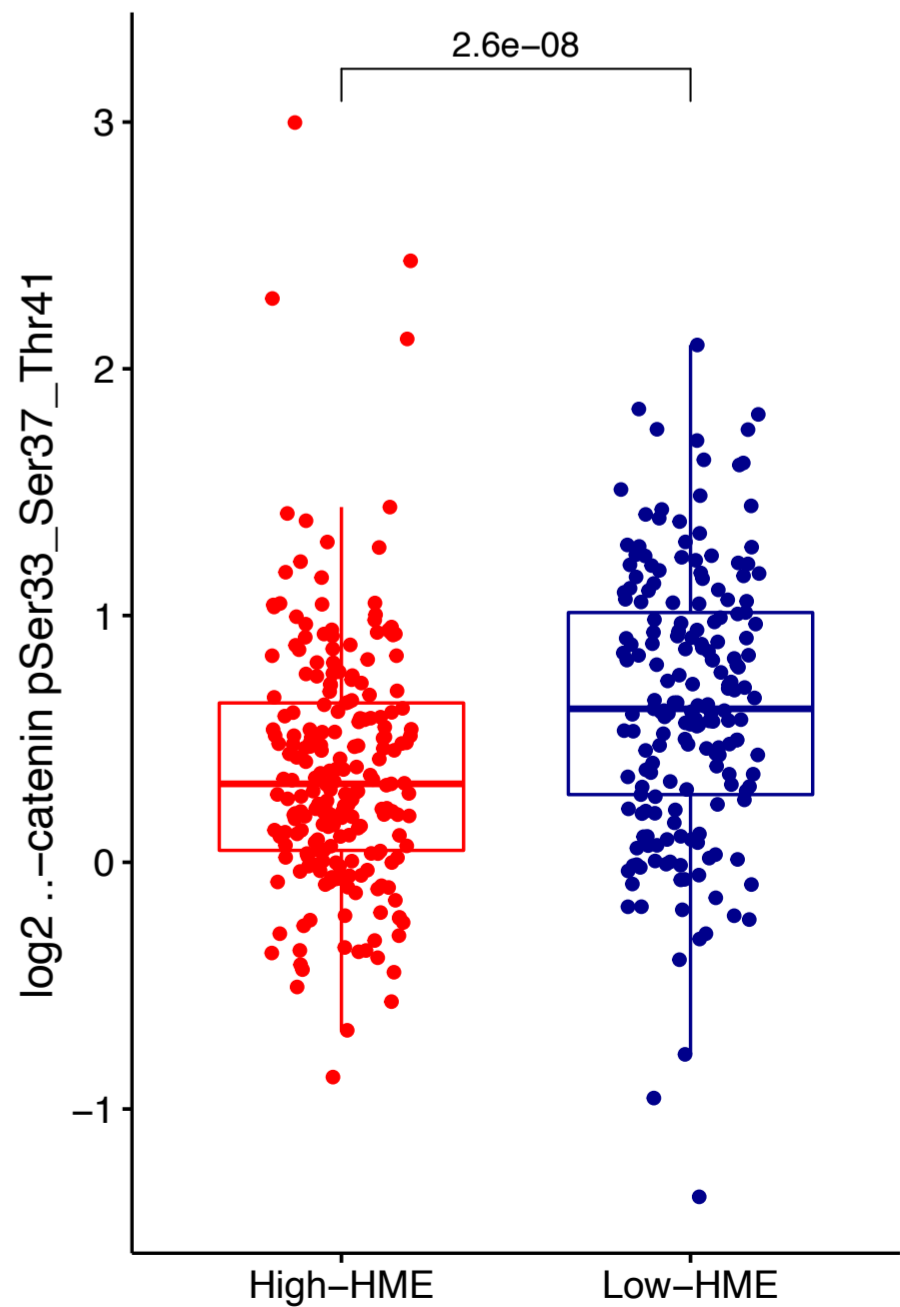

Supplement: Supplementary file 1 [file cancers-16-01448-s001.zip › Supplementary Figure S6.pdf]
