# Supplementary material for: Chromatin Profiles Are Prognostic of Clinical Response to Bortezomib-Containing Chemotherapy in Pediatric Acute Myeloid Leukemia: Results from the COG AAML1031 Trial
Source: Cancers (Basel). 2024 Apr 9;16(8):1448. doi: 10.3390/cancers16081448 (PMC11048007; doi:10.3390/cancers16081448)
Supplement: Supplementary file 1 [file Supplementary Figure S5.pdf › Supplementary Figure S5.pdf]

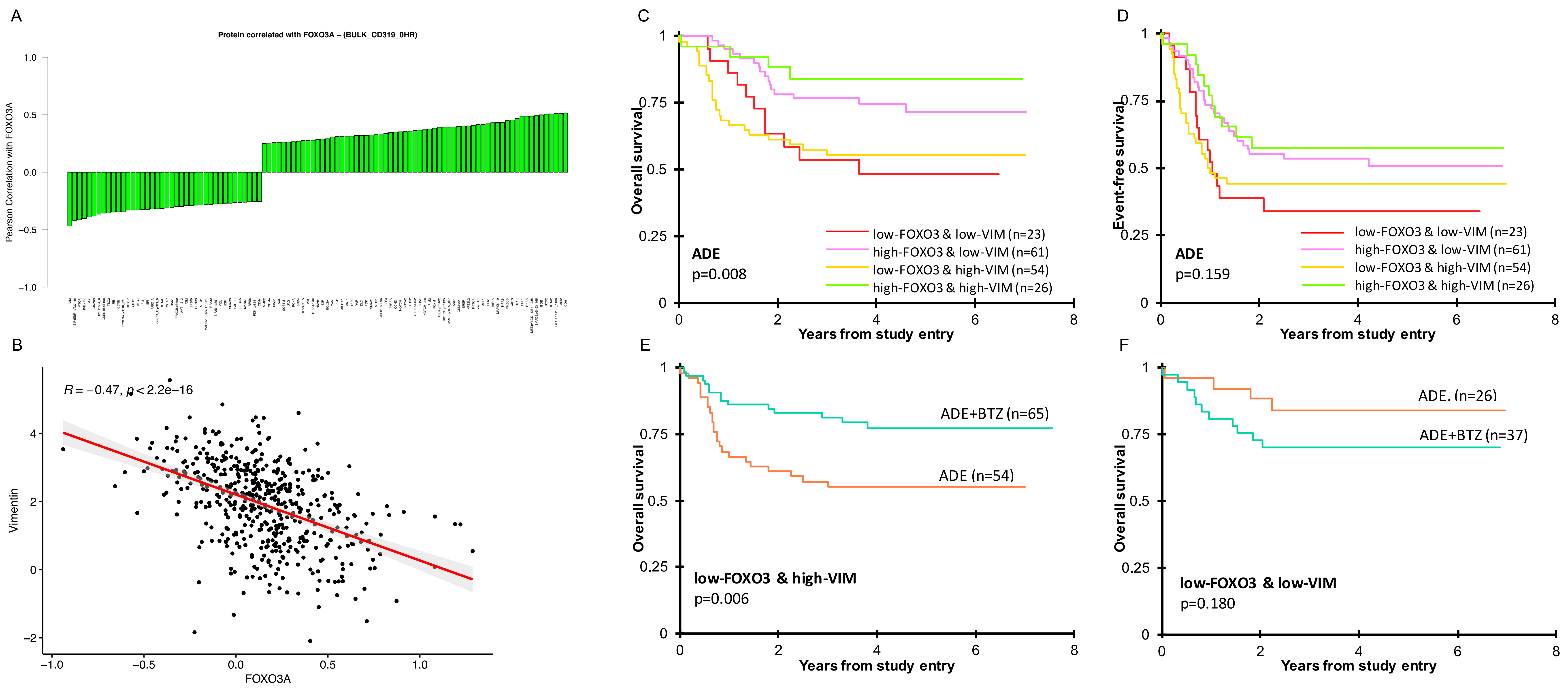
A
Protein correlated with FOXO3A – (BULK_CD319_0HR)
Pearson Correlation with FOXO3A
1.0
0.5
0.0
−0.5
−1.0
B
R = −0.47, p < 2.2e−16
Vimentin
FOXO3A
C
Overall survival
Years from study entry
ADE
p=0.008
low-FOXO3 & low-VIM (n=23)
high-FOXO3 & low-VIM (n=61)
low-FOXO3 & high-VIM (n=54)
high-FOXO3 & high-VIM (n=26)
D
Event-free survival
Years from study entry
ADE
p=0.159
low-FOXO3 & low-VIM (n=23)
high-FOXO3 & low-VIM (n=61)
low-FOXO3 & high-VIM (n=54)
high-FOXO3 & high-VIM (n=26)
E
Overall survival
Years from study entry
ADE+BTZ (n=65)
ADE (n=54)
low-FOXO3 & high-VIM
p=0.006
F
Overall survival
Years from study entry
ADE. (n=26)
ADE+BTZ (n=37)
low-FOXO3 & low-VIM
p=0.180
